# Decadal erosion of coral assemblages by multiple disturbances in the Palm Islands, central Great Barrier Reef

**DOI:** 10.1038/s41598-018-29608-y

**Published:** 2018-08-08

**Authors:** Gergely Torda, Katie Sambrook, Peter Cross, Yui Sato, David G. Bourne, Vimoksalehi Lukoschek, Tessa Hill, Georgina Torras Jorda, Aurelie Moya, Bette L. Willis

**Affiliations:** 1 College of Science and Engineering, James Cook University, Townsville, QLD 4811 Australia; 2 Australian Research Council Centre of Excellence for Coral Reef Studies, James Cook University, Townsville, QLD 4811 Australia; 3 Australian Institute of Marine Science, PMB 3, Townsville, MC, QLD 4810 Australia

## Abstract

Increases in the frequency of perturbations that drive coral community structure, such as severe thermal anomalies and high intensity storms, highlight the need to understand how coral communities recover following multiple disturbances. We describe the dynamics of cover and assemblage composition of corals on exposed inshore reefs in the Palm Islands, central Great Barrier Reef, over 19 years encapsulating major disturbance events such as the severe bleaching event in 1998 and Cyclone Yasi in 2011, along with other minor storm and heat stress events. Over this time, 47.8% of hard coral cover was lost, with a concomitant shift in coral assemblage composition due to taxon-specific rates of mortality during the disturbances, and asymmetric recovery in the aftermath thereof. High recruitment rates of some broadcast-spawning corals, particularly corymbose *Acropora* spp., even in the absence of adult colonies, indicate that a strong external larval supply replenished the stocks. Conversely, the time required for recovery of slow-growing coral morphologies and life histories was longer than the recurrence times of major disturbances. With interludes between bleaching and cyclones predicted to decrease, the probability of another severe disturbance event before coral cover and assemblage composition approximates historical levels suggests that reefs will continue to erode.

## Introduction

Decadal declines in coral cover have been reported across all ocean basins^1–3^. Drivers of coral loss range from localised stressors, such as overfishing and poor water quality^4,5^, to large-scale disturbances such as coral bleaching, crown-of-thorns starfish, disease outbreaks and cyclones^6^. Episodic disturbances form a natural component of the dynamics of coral reef ecosystems^7,8^ and play an important role in structuring benthic community composition^9–11^. However, under current climate change scenarios, predicted increases in the frequency and intensity of coral bleaching and severe storms^12–15^ raise serious concerns about the long-term persistence of coral reefs, their resilience to multiple disturbances, and potential for future recovery^4,10,16–19^.

Many studies examining the effects of disturbance on coral reefs have used coral cover as a proxy for recovery^3,9,20,21^. Although assessing the return of coral cover to pre-disturbance levels provides a rapid mechanism for evaluating recovery over relatively large spatial scales, measuring coral cover alone may obscure more serious changes to underlying ecosystem functions^22–26^. Incorporating additional coral recovery metrics, such as taxonomic composition and population demographics into survey designs, can offer greater resolution about recovery patterns following disturbance^27^. Such data can provide insights into whether the benthic community is re-assembling to the pre-disturbance composition^26^ and identify potential limitations to recovery^28^. Unsuitable settlement substrata^29^, changes to localised hydrodynamic regimes^22^, absence of broodstock^30^, and limited external larval supplies^31^ can restrict recruitment success and impede recovery, while post-settlement mortality can be high under unfavourable conditions for growth^32,33^. Importantly, differences in life history traits among species typically result in differential recovery rates and consequently a ‘recovered’ reef may have a dissimilar composition compared to the assemblage prior to disturbance^31^.

**Figure 1:**
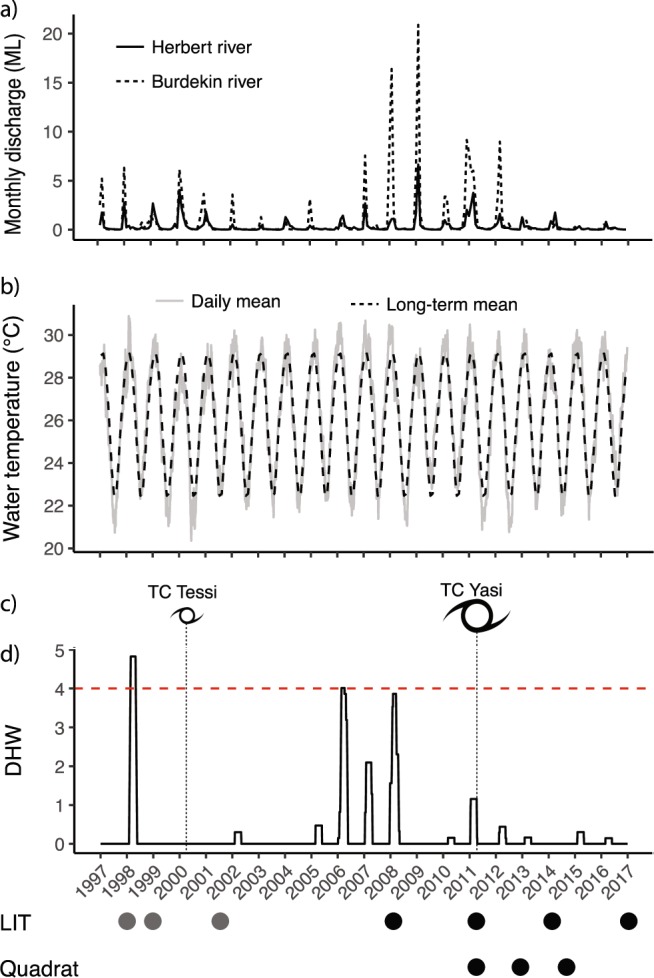
Disturbance history of fringing reefs of the Palm Islands, central Great Barrier Reef, between 1997 and 2017, in relation to sampling time points (LIT = Line Intercept Transect; grey circles represent historical data^46^, black circles represent new data collected for the present study). (**a**) Monthly discharge of Herbert (solid line) and Burdekin (dashed line) rivers in megalitres (Source: Queensland Government Water Monitoring Portal, https://water-monitoring.information.qld.gov.au/host.htm); (**b**) daily mean (solid grey line) and long-term monthly mean (dashed line) water temperature (in °C) at Orpheus Island (Source: Australian Institute of Marine Science Historical Data Tool, http://data.aims.gov.au/aimsrtds/datatool.xhtml); (**c**) timing of two cyclones affecting the Palm Islands between 1997 and 2017 (Cat. 2 TC Tessi in Apr 2000, http://www.bom.gov.au/cyclone/history/tessi.shtml; Cat. 5 TC Yasi in Feb 2011, http://www.bom.gov.au/cyclone/history/yasi.shtml); (**d**) cumulative heat stress on reefs of Orpheus and Pelorus Islands (in Degree Heating Weeks, DHW) calculated from temperature data presented in panel C, following NOAA’s protocol^80^; dashed red line indicates the theoretical bleaching threshold of 4 DHW.

Until recently, cyclones were estimated to be responsible for one-third^6^ to almost one-half^3^ of the decline in coral cover on the Great Barrier Reef (GBR) over the past three decades, while bleaching was estimated to have caused between 5.6% to 10% of loss in coral cover^3,6^. However, in 2016 alone, 30% of the shallow-water corals died over the entire GBR^34^, which was further exacerbated by a subsequent bleaching event in 2017 (damage yet unquantified), highlighting the increasing contribution of bleaching to reef degradation. Fringing reefs of the Palm Island Group, located on the inner shelf of the central section of the GBR (Supplementary Fig. S1) were heavily impacted by the first reported mass bleaching event on the GBR in 1998^35^. Susceptible coral taxa suffered 100% mortality at some locations in the Palm Islands^36^, and heat stress negatively impacted the reproductive potential of surviving populations^37^. Thirteen years later, in February 2011, Cyclone Yasi, one of the largest and most severe cyclones to affect the region^38^, tracked a path just to the north of the Palm Island Group, packing maximum wind speeds up to 285 km/hr, with sustained winds up to 205 km/hr^39^. Post-cyclone surveys of mid- and outer-shelf reefs indicated widespread and significant damage, although impacts on fringing reefs of the Palm Island Group differed strongly between leeward and windward locations^21,40^. The 2016 bleaching event was most severe in the northern GBR, and left the Palm Island Group largely unaffected^14^ (Fig. 1). In the 13 years between the bleaching event and Cyclone Yasi (1998–2011), and in the six years after the cyclone (March 2011 – February 2017, i.e. up until the 2017 bleaching event), there have been only minor disturbances to reefs in the Palm Island Group^21,41–43^ (Fig. 1), including a category 2 cyclone and minor heat anomalies that were insignificant in comparison to the 1998 and 2011 events (Fig. 1). These two interludes provide an opportunity to examine the immediate impact of different acute disturbances (mass bleaching versus category 5 Cyclone Yasi), and to follow recovery trajectories of coral assemblages at affected sites. Here we document changes to benthic communities over time on exposed fringing reefs of Orpheus and Pelorus Islands, in the Palm Island Group, to compare and evaluate the impact of these two major disturbances on live coral cover and benthic community composition. We also quantify the nature of cyclone damage to these reefs, describing fine-scale recovery patterns with high taxonomic and spatial resolution.

## Methods

### Study sites

Data were collected from fringing reefs at two windward sites; one on the north-eastern side of Orpheus Island (OI) and the second on the eastern side of Pelorus Island (PI) in the Palm Island Group. Each site was severely impacted by the 1998 mass bleaching event^44^, and in the direct path of severe tropical Cyclone Yasi in 2011 (Supplementary Fig. S1).

### Benthic community composition from 1998 to 2017

Benthic composition at each site was quantified by visual census using three replicate 20 m line intercept transects (LITs) on exposed reef crests (2–3 m below chart datum) in June 2008, November 2011, December 2014 and March 2017 (Fig. 1). The substrate immediately beneath the transect tape was recorded and the intercept length measured to the nearest centimeter. All live hard corals were identified to genus, and *Acropora* spp. were further classified into the following morphological categories: bottlebrush, bushy, clumping, corymbose, digitate, arborescent, and tabular; following Veron^45^. Soft corals were pooled into one category.

For comparison with coral cover and assemblages immediately following the 1998 bleaching event, data for 1998, 1999 and 2001 were extracted from Gralton (2002)^46^. These data were collected at the same sites and reef zones using the identical line-intercept methods. However, corals were identified only to the level of four gross morphotypes: branching/arborescent, corymbose/plating, encrusting and massive^46^, and results presented as mean ± SE for the two sites combined. In order to compare these earlier data with data collected post-2008, the more recently collected data were re-classified into these four morphotypes following Veron^45^ (Supplementary Table S1). As the raw data were not available for 1998, 1999 and 2001, they could not be included in formal statistical analyses.

Trends in overall and morphology/taxon-specific cover (decline and recovery rates 1998–2017) were visualized on mean ± SE. Taxon-specific trends in coral cover post-2008 were assessed using a generalized linear mixed-effects model in a Bayesian framework, with binomial distribution, where the number of ‘successes’ is the total number of centimeters a coral taxon occupies under the transect line, and the number of ‘failures’ is the total number of centimeters a coral taxon is not found under the transect line. Time-points with only zero values (2011 for *Acropora* and 2014 for Poritidae) were excluded from analyses. Temporal changes in the taxonomic composition of benthic communities following Cyclone Yasi were visualized using non-parametric multidimensional scaling (nMDS) using a Bray-Curtis dissimilarity matrix. Cover of each coral category in each transect was fourth-root transformed, and standardized by the respective maximum value for each coral category across all transects (‘column maxima’). In order to retain contrasts between rare and abundant coral categories, we did not standardize by the maximum cover of all coral categories per site (i.e., by ‘row maxima’). The similarity percentages routine (SIMPER) was used to examine which coral genera were driving differences in taxonomic composition among time-points and a pairwise permutational MANOVA on the Bray-Curtis dissimilarity matrix was used to identify significant differences among years.

### Demographic dynamics of corals following Cyclone Yasi

In July 2011, five months post Cyclone Yasi, six permanent 5 × 5 m quadrats were established on the OI site (three replicate quadrats in each of two zones: reef crest and reef slope - 4–5 m below chart datum) to monitor the dynamics of coral populations at fine spatial scales and high taxonomic resolution. Quadrats within zones were spaced ~10 m apart and surveyed in July 2011, November 2013 (two years and nine months post-Cyclone Yasi) and January 2015 (almost four years post-cyclone) (Fig. 1). Within each quadrat, each hard coral colony ≥1 cm was identified to genus. Mean colony diameter was estimated for each colony using the mean of the maximum colony diameter (measured to the nearest cm) and the diameter measured at right angles. Published linear growth rates for *Acropora* (4.6–12 cm/yr^47^), *Montipora* (2–5 cm/yr^48^), *Pocillopora* (4.32 cm/yr^49^), Faviidae (0.17–1.27 cm/yr^50^) and Poritidae (0.13–2.21 cm/yr^51^) were used to infer which colonies were likely to have been present prior to Cyclone Yasi and which had recruited post-cyclone. Logistical constraints and inclement weather prevented re-survey of one reef slope quadrat in 2013.

To account for the three-dimensional nature of the substratum and concomitant potential to bias calculations of coral colony density^52^, a structural complexity correction factor was applied to each 5 × 5 m quadrat to calculate its true area. Each quadrat was subdivided into twenty-five 1 × 1 m “mini-quadrats”. Within each mini-quadrat, rugosity and structural complexity were estimated visually^53^, and assigned a value based on a 6-point scale ranging from 1.0 (low topographic complexity) to 1.6 (high topographic relief). The mean complexity index value of mini-quadrats was used as a correction factor for the quadrat. The true area of each quadrat was calculated by multiplying 25 m^2^ by the quadrat’s correction factor.

Temporal changes of colony density, coral cover and diversity (at genus level) in each zone were tested in Bayesian generalized linear mixed-effects models, using negative binomial, binomial and poisson distributions, respectively (Supplementary material); with an interaction between year and zone, and quadrats as varying coefficients (equivalent to random effects). We performed various contrasts to ask specific questions, calculated the effect size from the posteriors and determined the probability that the effect size was greater than zero. We report 95% credibility intervals (CI) and the probability of the posterior of these contrast (P). This P value is not the same as the frequentist ‘p-value’; higher P values reflect higher probability, hence are similar to lower p-values.

All data manipulation and statistical analyses were carried out in R^54^ (list of packages, references and scripts in Supplementary material).

## Results

### Changes in overall coral cover: 1998–2017

**Figure 2:**
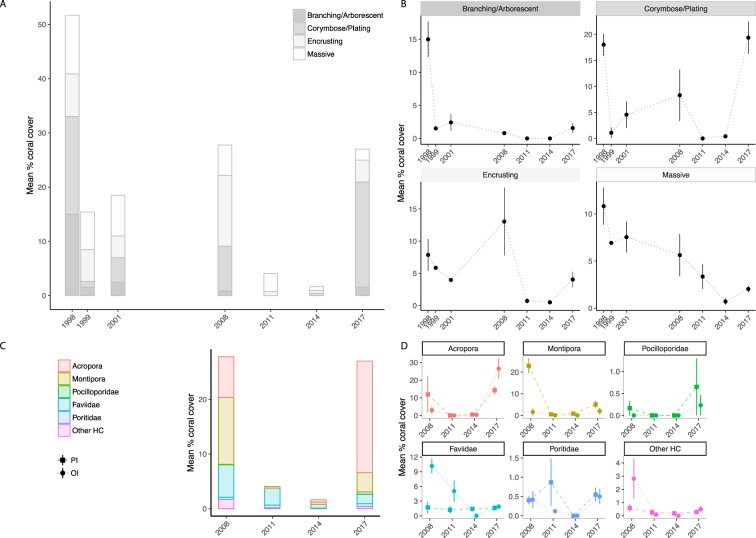
Mean percent cover of four hard coral morphologies at exposed reef crests of Orpheus and Pelorus Islands, central GBR, from before the 1998 mass bleaching event, through the 2011 cyclone event, until 2017 (**A**,**B**). The same data grouped taxonomically for the period 2008–2017 (**C**) and presented separately for Orpheus (OI) and Pelorus (PI) Islands (**D**). Data from 1998, 1999 and 2001 were obtained from Gralton (2002). All datasets (1998–2014) were obtained from three replicate 20 m line intercept transects per island per time-point. Error bars on point graphs represent SE.

Hard coral cover (HCC) decreased by 47.8% between 1998 and 2017 at the exposed fringing reefs of Orpheus and Pelorus Islands (Fig. 2A). This timeframe encapsulates two episodes of coral decline and recovery. The first was the 1998 bleaching event, during which live coral cover dropped from 51.7% to 15.4% followed by a roughly decade-long recovery period with few minor disturbances^41–43^ (Fig. 1) during which coral cover increased to 27.8% (Fig. 2A). The second was the 2011 Cyclone Yasi event, which reduced live coral cover to 4.1%, followed by ongoing decline to 1.6% in 2014, after which recovery began and coral cover increased to 27.0% by 2017 (Fig. 2A). The average rate of recovery of hard coral cover during the decade-long interlude following the bleaching event was 1.4% year^−1^ (HCC_1999_ = 15.4%, HCC_2008_ = 27.8%) (Fig. 2A). The rate of increase in coral cover in the first three years following the bleaching event (1998–2001, despite an intervening category 2 cyclone in 2000) was almost identical to the rate of increase during the subsequent seven years (2001–2008, which included a minor heat stress event in 2006) (Figs 1 and 2A). In contrast, hard coral cover continued to decline during the first three years following Cyclone Yasi in 2011; however, there was a subsequent rapid increase in coral cover from 2014 to 2017, with a mean recovery rate of 8.5% year^−1^. With this recovery rate, the overall coral cover in 2017 has almost reached the 2008 pre-cyclone levels (HCC_2008_ - HCC_2017_ = 0.8%, CI: −0.7–2.5%, P = 0.8).

### Changes in assemblage composition: 1998–2017

The cover of branching/arborescent and corymbose/plating corals (predominantly *Acropora* spp.) decreased by 89.8% and 93.9%, respectively, between 1998 and 1999 as a consequence of bleaching (Fig. 2A,B). In comparison, losses of encrusting and massive corals were only 25.5% and 36.0% of cover, respectively. The category 5 cyclone caused 100% loss in cover of branching/arborescent and corymbose/plating corals, 94.5% loss in the cover of encrusting corals, and 40.3% in the cover of massive corals. (Fig. 2A,B). Interestingly, even in the absence of major acute disturbances during the decade between the bleaching event and Cyclone Yasi^41^, with the exception of corymbose/plating corals, the cover of all growth forms suffered periods of decline (Fig. 2A,B). For example, the cover of encrusting corals decreased a further 32.0% in the first two years after the 1998 bleaching (between 1999 and 2001, a period which included the category 2 Cyclone Tessi, Fig. 1), while the cover of branching/arborescent and massive growth forms declined by 66.9% and 22.4%, respectively, between 2001 and 2008 (which encompassed a mild thermal stress event, Fig. 1; Fig. 2A,B). The dominant growth form shifted from corymbose/plating corals before the bleaching event, to massive and encrusting corals following the bleaching event, and until six years after the cyclone event, when corymbose/plating corals dominate the assemblage once again (Fig. 2A,B). Importantly, by 2017 corymbose/plating corals exceeded and encrusting corals started to approximate the 1998 levels of cover, while branching/arborescent and massive corals remain at low cover levels.

The detailed taxonomic analysis of assemblage composition revealed that the post-cyclone recovery was mainly driven by *Acropora* species (Fig. 2C,D, Supplementary Fig. S2). The cover of faviids and poritids continued to decline following the cyclone (lag-effect; between 2011 and 2014). Cover of every coral category increased in the period three to six years post-cyclone, with an order of magnitude difference in the rate of recovery of *Acropora* spp. compared to other taxa. Pocilloporid species recovered in distinct patches, indicated by the large error bars in 2017 (Fig. 2D).

**Figure 3:**
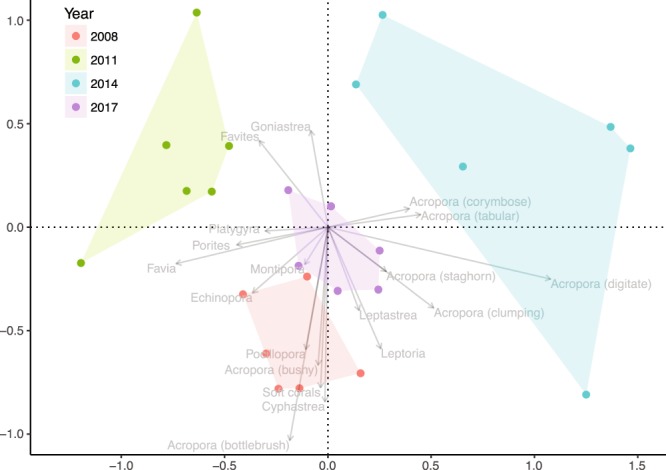
Non-parametric MDS ordination based on coral assemblage composition by year at exposed reef crests of Orpheus and Pelorus Islands on the central GBR. Genus-level coral cover data on six replicate 20 m line intercept transects on the reef crest were fourth-root transformed and standardized by column maxima. 2D stress: 0.16.

Coral assemblages formed distinct clusters by year (pairwise permutational MANOVA, all pairwise comparisons between years p < 0.05; Fig. 3), with a significant shift in the composition from a high diversity assemblage in 2008, to a low diversity assemblage dominated by massive corals after the cyclone in 2011. In 2014 (three years post-cyclone) there was substantial variability among transects, with the main direction of shift driven by an increased cover of *Acropora* spp. and decreased cover of massive corals (c.f. lag effect, above). By 2017 the variability decreased and the resulting assemblage was significantly different from both the pre- and immediate post-cyclone assemblages (pairwise permutational MANOVA, p < 0.05; Fig. 3).

### Demographic dynamics of corals following Cyclone Yasi

**Figure 4:**
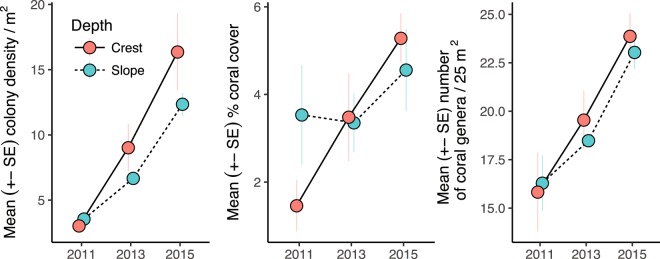
Mean + - SE colony density (**a**), coral cover (**b**), genus diversity (**c**) by depth and year on the exposed fringing reef of Orpheus Island, central GBR. Data obtained from three replicate 5 × 5 m quadrats per depth zone per time-point (bar the slope in 2013, when only two quadrats were surveyed for logistical constraints).

In July 2011, five months after Cyclone Yasi, a total of 606 coral colonies (≥1 cm mean diameter) were counted in the six permanent quadrats on the reef crest and slope of Orpheus Island. By January 2015, the number of colonies had increased five-fold to 3,109. On average, in 2011 reef crest quadrats had 14 fewer colonies than reef slope quadrats (CI: -73–48, P = 0.7) whereas in 2015 reef crest quadrats had 102 more colonies than quadrats on the reef slope (CI: -153–375, P = 0.8 (Fig. 4, Supplementary Fig. S3, Supplementary Tables S3 and S4). These differences translate to 43% (CI: -35–129%) faster net increase in abundance on the reef crest compared to the reef slope. Similarly, in 2011 coral cover on the crest was 48% of coral cover on the reef slope (CI: 9–97%, P = 0.9) but was 1.3 times higher by 2015 (CI: 0.3–2.6, P = 0.7), translating to more than five times faster net growth of cover on the reef crest than slope (CI: 0.2–20.2, Fig. 4, Supplementary Fig. S3, Supplementary Tables S3 and S4). Genus diversity did not differ significantly between the crest and the slope at any time-point (Fig. 4, Supplementary Fig. S3, Supplementary Tables S3 and S4), but increased between subsequent time-points (P_2011–2013_ = 0.8, P_2013–2015_ = 0.9). These patterns were largely driven by a disproportionately high recruitment of *Acropora* and *Montipora* on the reef crest in 2013 and 2015 (Supplementary Fig. S4), and a higher mortality of large *Montipora*, *Porites* and *Platygyra* colonies on the reef slope (Supplementary Fig. S5).

**Figure 5:**
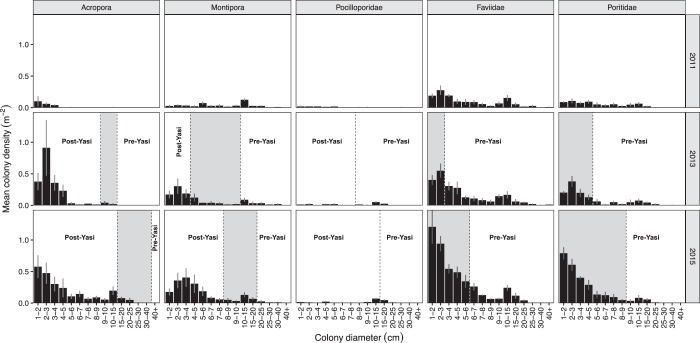
Size frequency distribution of coral groups based on mean colony diameter (cm) in 2011, 2013 and 2015. Dashed lines indicate the minimum size of predicted survivors of Cyclone Yasi based on published linear growth rates^3–6^. The gray shaded areas indicate colony size classes that may include survivors of Yasi or post-Yasi recruitment, due to variability in growth rates.

In 2011, mean colony size distributions of *Acropora* and Pocilloporidae (branching, plating and corymbose morphologies) were skewed towards small colonies, whereas more balanced size frequency distribution was observed for encrusting and massive colonies of *Monitpora*, faviids and poritids (Fig. 5). In subsequent years the dominant demographic pattern was an increase in small size classes of broadcast spawning species (*Acropora*, *Montipora*, Faviidae and Poritidae); contrasted by a slow recruitment of brooding pocilloporid species (Fig. 5).

## Discussion

Over the last two decades, the fringing reefs of Orpheus and Pelorus Islands in the central GBR have been impacted by two major and several minor disturbances. These disturbances have resulted in substantial declines in coral cover and a shift in benthic community composition, including alterations in the taxonomic makeup of coral assemblages^21^. Uneven mortality rates among coral taxa and growth forms during and immediately after the mass bleaching event in 1998 and severe Cyclone Yasi in 2011 led to rapid shifts in the composition of coral assemblages^21^. Slow growing massive corals, which are generally more robust to both thermal stress^36^ and physical disturbance^55,56^, disproportionately dominated coral assemblages on exposed reefs immediately following the 1998 bleaching and the 2011 cyclone events. Similar changes in coral assemblages following perturbations have been reported previously, with slow growing massive coral colonies commonly dominating post-disturbance assemblages^25,29,34^. However, although post-disturbance mortality was less severe for massive and encrusting corals compared to other growth forms, their cover continued to decline during the three subsequent years. Thermal stress and physical injury have both been shown to trigger coral diseases^57–59^, which is a plausible pathway to the delayed mortality among survivors of the acute stress in our study. Additionally, the disruption of the intricate ecological feedback loops that exist among corals, their competitor algae, their predators and myriads of species of reef fish can cause further mortality in the aftermath of the disturbance, and retard recovery. For example, the increased per capita predation of corals by *Drupella* snails and Crown-of-Thorns starfish has been observed to further decimate coral colonies that survived cyclones and bleaching (pers. obs.; Hughes *et al. in review Nat. Clim. Change*). Algae that often bloom in the aftermath of acute disturbances on the reef can impede coral recruitment^60^, which can lead to large-scale failure in recruitment and even ecosystem collapse^61^. Importantly, a recent study has shown that increasing chronic stressors (rising sea temperatures, decreasing water quality) have an adverse effect on coral recruitment and growth rates, suggesting further lags in coral recovery in the future^62^. Regardless of the underlying mechanisms, such lag effects can have severe consequences for the dynamics of coral assemblages, particularly for the subsequent recovery of populations of slow growing massive corals, yet they are rarely considered in assessments of ‘winners and losers’ of perturbations on tropical coral reefs. Importantly, the loss of large colonies of slow growing species can leave a footprint on the assemblage composition for many decades^14^, if not for centuries.

### Taxonomic differences in recovery

The effects of differential mortality among coral taxa, overlaid by taxon-specific rates of recruitment and growth rates created novel coral assemblage configurations in the aftermath of both major disturbances. Almost two decades after the 1998 bleaching event, only corymbose and plating corals approximated historic levels of cover. Branching corals (mainly consisting of *Acropora* spp.) that were historically abundant on the study reefs, failed to show any sign of recovery in the study period. Because the reproductive strategies, and hence the larval dispersal potential of branching and corymbose/plating *Acropora* species are similar^63,64^, the lack of recovery in the former but almost complete population recovery in the latter suggests that the respective source populations could have been affected differently. Indeed, arborescent *Acropora* species have been ranked as the most sensitive group of corals under extreme heat anomalies^14^, and it is likely that their populations suffered declines at a wider geographic footprint in 1998 than did the populations of other, less sensitive *Acropora* species. The lower rates of recruitment of non-acroporid corals may also be due to larval limitation, or lower settlement and post-settlement survival rates than acroporids. Some coral taxa, such as poritids, faviids and isoporan acroporids are known to be poor recruiters, even when adult colonies are abundant^65^.

For corals with low gamete or larval dispersal rates, density-dependent mechanisms (e.g. reliance on strong self-seeding) may hinder population recovery. For example, brooded larvae of pocilloporids are capable of settling immediately upon release, potentially limiting their dispersal range^66^. In accordance, with low numbers of mature corals surviving the cyclone, we observed only a small increase in the abundance of pocilloporids between 2011 and 2014 in permanent quadrats, and the increase in their cover is attributed to the growth of the few colonies surviving Cyclone Yasi. Admittedly, the lack of taxon-specific information prior to the 1998 bleaching event precludes direct comparisons with historic pre-disturbance population sizes. In contrast to brooding pocilloporids, sympatric broadcast spawning *Acropora* spp. with similar colony morphologies showed rapid recovery, even in the absence of reproductively mature colonies, demonstrating the presence of a strong external source of larvae for broadcast spawners^40^, but not for brooders. This highlights the critical importance of maintaining connectivity with undisturbed stocks^22,30,67^ for effective population recovery, as well as the importance of understanding stock-recruitment relationships and likely limitations to recovery imposed by corals with brooding life histories^22^. Limited capacity to recover when adult stocks of brooding corals are depleted may result in local extirpations – a concern that may have already been realized for *Seriatopora hystrix* on reefs in the inner Seychelles^25^.

The dependence of brooding corals on local broodstock for population maintenance and recovery, and low recruitment rates of poritids and faviids, even when larvae were presumably available^65^, accentuates their higher vulnerability to extreme disturbances in the long term because their populations may take longer to bounce back to pre-disturbance levels than populations of fast growing, good recruiters, such as *Acropora* and *Montipora spp*. Importantly, a modeling study has shown that even fast-growing *Acropora* populations on inshore reefs of the GBR are likely to lose resilience at a sustained or increasing frequency and severity of disturbance events^68^.

### Spatial and temporal patterns in recovery

With a 1.4% annual increase in coral cover in the decade following the 1998 bleaching event (estimated from historical data^46^), and assuming a similar linear increase over future time periods, the fringing reefs of the Palm Islands would have taken over 20 years to return to pre-1998 levels. Cyclone Yasi interrupted the recovery of reefs on exposed windward sides of the islands but triggered a substantially faster rate of recovery in the aftermath. A substantial increase in newly colonized coral colonies was recorded at the Pelorus Island study site over 5 years post Cyclone Yasi^27^. The immediate impact of cyclones is typically to open up space for recolonization in an often space-limited ecosystem^11^. Our study sites were not space limited before the cyclone (e.g. they had low coral cover and were not overgrown by macroalgae^21^), although it is possible that the epilithic algal matrix or biofilm impeded recruitment over the 1998–2008 period, which was then removed by the mechanical forces associated with the cyclone. Alternatively, the integrity of coral broodstocks may have been compromised over large areas by the 1998 bleaching event, which could have led to diminished larval supply and hence recruitment failure over multiple years^69^. Recent population genetic studies identified limited cross-shelf connectivity among coral populations south of 19°S^70^, suggesting that the source populations of demographic rescue on the Palm Islands are mainly other inshore reefs. However, in 1998 bleaching was most severe on inshore reefs from Heron Island (23°26′ S, 151°55′ E) to Elford Reef (16°50′ S, 146°13′ E)^35,44^, and surviving coral colonies were reported to suffer decreased fecundity over the spawning seasons following the heat stress^37^. In this scenario, coral populations outside the impact zone of Cyclone Yasi, but previously affected by the 1998 bleaching event, may have reached the maturity to supply larvae in abundance to downstream reefs in the years following the cyclone. Finally, the moderate disturbances during the period between the mass bleaching event and TC Yasi could have negatively affected recovery. For example, the category 2 cyclone Tessi in 2000 did not cause excessive winds and waves in the area, but brought about increased rainfall and terrestrial runoff, possibly causing physiological stress and increasing disease susceptibility in corals, which would explain the reduction of encrusting corals (mainly *Montipora spp*., pers. obs.) on our study sites.

We found that the most rapid recovery of coral assemblages on reef crests coincided with the highest rates of cyclone damage, suggesting that these shallow habitats are naturally more dynamic. Coral cover and density were lower on the reef crest than the reef slope five months after TC Yasi, and yet both indices increased more steeply on shallow crests than on slopes in the following four years. Impacts of unusually intensive perturbations, such as category 5 tropical cyclones or extreme bleaching events, may operate over an extended depth range, damaging less dynamic slope habitats, and potentially causing long lasting effects. Interludes between disturbances are predicted to decrease as climate change progresses^71,72^, which means reef slope communities are expected to suffer more pronounced alterations than the naturally dynamic reef flat and reef crest habitats.

### Prospects of complete reef recovery

The overall coral cover in 2017 had not yet reached the pre-cyclone levels but based on current recovery rates it could be estimated to exceed that by 2018, i.e. seven years post-disturbance. This is on a par with the roughly decade-long period estimated for recovery from perturbations in other studies^24,26,30,73–75^. These promising figures are, however, misleading, if we consider that (i) the pre-cyclone coral cover was already approximately half of the known historical levels (i.e., in 1998), which in turn was most likely a non-pristine state itself (shifting baselines^76^); (ii) the composition of the coral assemblage has also drastically changed since 1998, with slow growing massive corals experiencing alarming declines, and arborescent corals, as well as pocilloporids failing to recover; (iii) recovery rates are predicted to decrease in the future as climate change progresses^62^; and (iv) our 2017 data were collected at the onset of yet another thermal stress event, stronger than the 2016 anomaly at our survey sites (Hughes *et al*. in review Nat. Clim. Change). The increase in frequency and severity of acute disturbances due to weather extremes associated with global climate change^12–15,71,72^ suggests that the exposed fringing reefs of Orpheus and Pelorus Islands, along with many other coral reefs in the world, may never recover to pre-disturbance conditions, and will continue to erode. Coral species each perform important functions on the reef, by providing food, shelter and creating habitat, that in turn determine the composition of the reef community with critical feedback loops for the persistence of corals^11,77,78^. For example, the interstitial space characteristic of each coral species provides unique micro-habitats that together underpin the diversity of reef ecosystems^79^. The lack of recovery in certain coral taxa can therefore trigger unpredictable cascades of functional changes in the ecosystem.

## Electronic supplementary material

Supplementary information

Dataset 1

Dataset 2

Dataset 3
