# Supplementary material for: Decadal erosion of coral assemblages by multiple disturbances in the Palm Islands, central Great Barrier Reef
Source: Sci Rep. 2018 Aug 8;8:11885. doi: 10.1038/s41598-018-29608-y (PMC6082856; doi:10.1038/s41598-018-29608-y)
Supplement: Supplementary file 1 — Supplementary information [file 41598_2018_29608_MOESM1_ESM.docx]

## Supplementary material for

**Decadal erosion of coral assemblages by multiple disturbances in the Palm Islands, central Great Barrier Reef**

Gergely Torda^1, 2, 3*^, Katie Sambrook^1, 2*^, Peter Cross^1^, Yui Sato^3^, David G. Bourne^1, 3^, Vimoksalehi Lukoschek^2^, Tess Hill^1^, Georgina Torras Jorda^1^, Aurelie Moya^2^, Bette L. Willis^1, 2^

* equal contribution

1. College of Science and Engineering, James Cook University, Townsville, QLD 4811, Australia

2. Australian Research Council Centre of Excellence for Coral Reef Studies, James Cook University, Townsville, QLD 4811, Australia

3. Australian Institute of Marine Science, PMB 3, Townsville MC, QLD 4810, Australia

Email: gergely.torda@jcu.edu.au

**
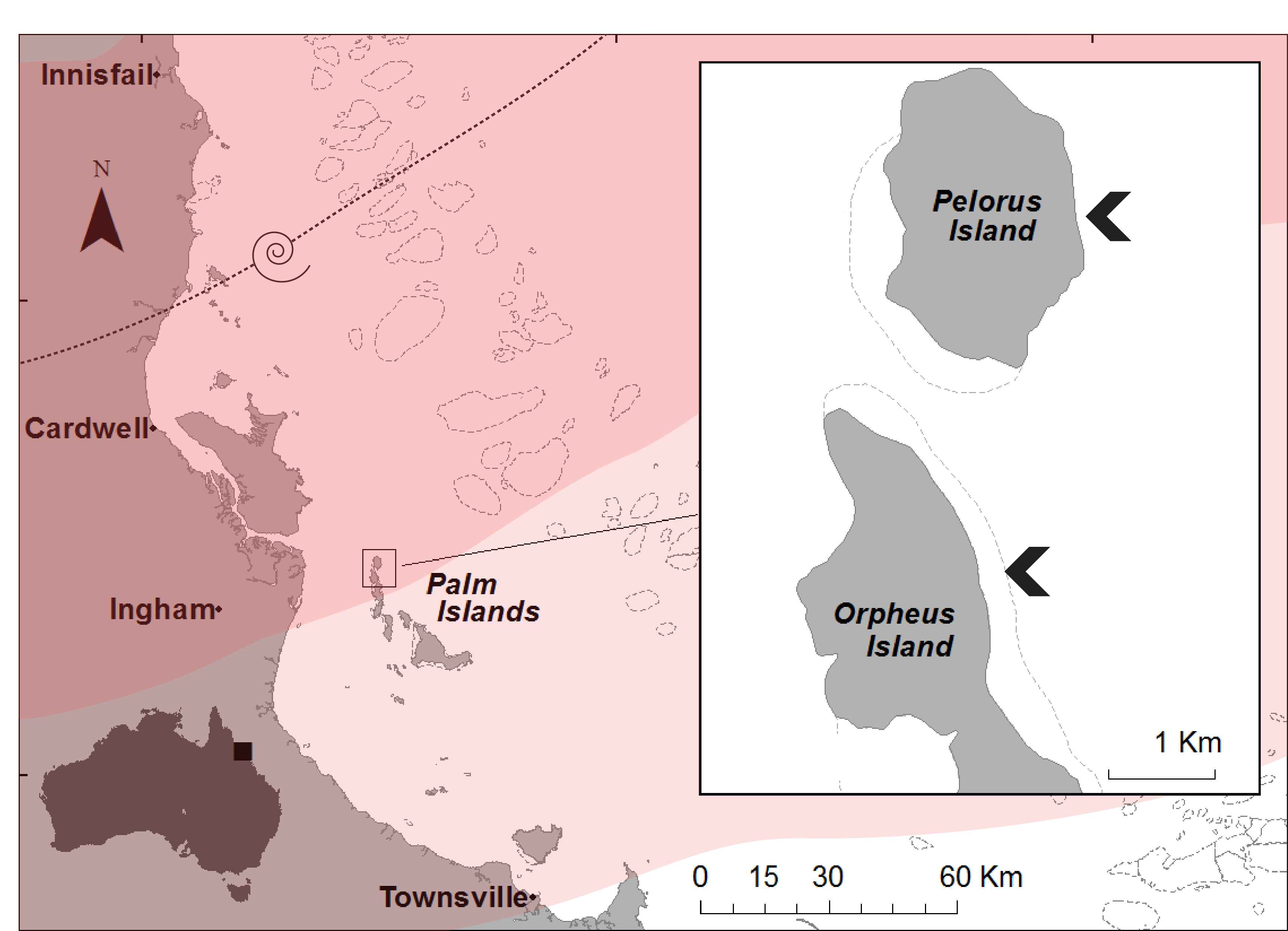
**

**Fig. S1** Map showing the location of survey sites and the track of Category 5 Tropical Cyclone Yasi (dashed line) as it travelled past the Palm Islands Group, in the central Great Barrier Reef, on 2 February 2011. Strong pink shading indicates boundaries of very destructive winds with gusts exceeding 280 km/h; light pink shading indicates boundaries of destructive winds with gusts between 125 and 280 km/h. Map created in ArcGIS 10, cyclone track data extracted from http://www.bom.gov.au/cyclone/history/yasi.shtml.


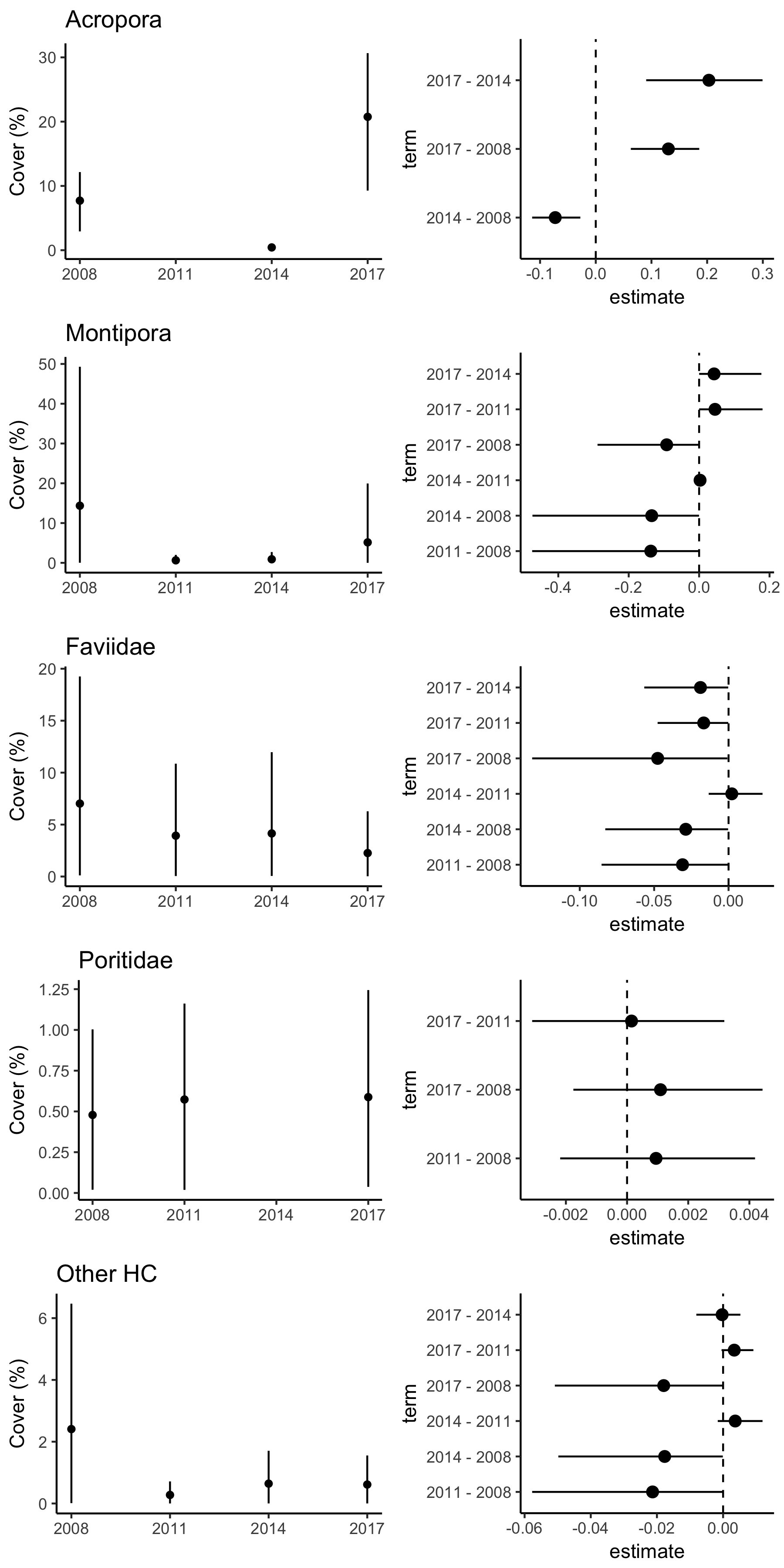


**Fig. S2** Means and 95% credibility intervals of modeled coral cover for five coral groups obtained from Bayesian generalized linear mixed effects models using binomial distibuiton. Years with only zero values were omitted (2011 for *Acropora* and 2014 for Poritidae). Corresponding butterfly-plots indicate the 95% prediction intervals of pairwise comparisons of timepoints, where zero represents no change.


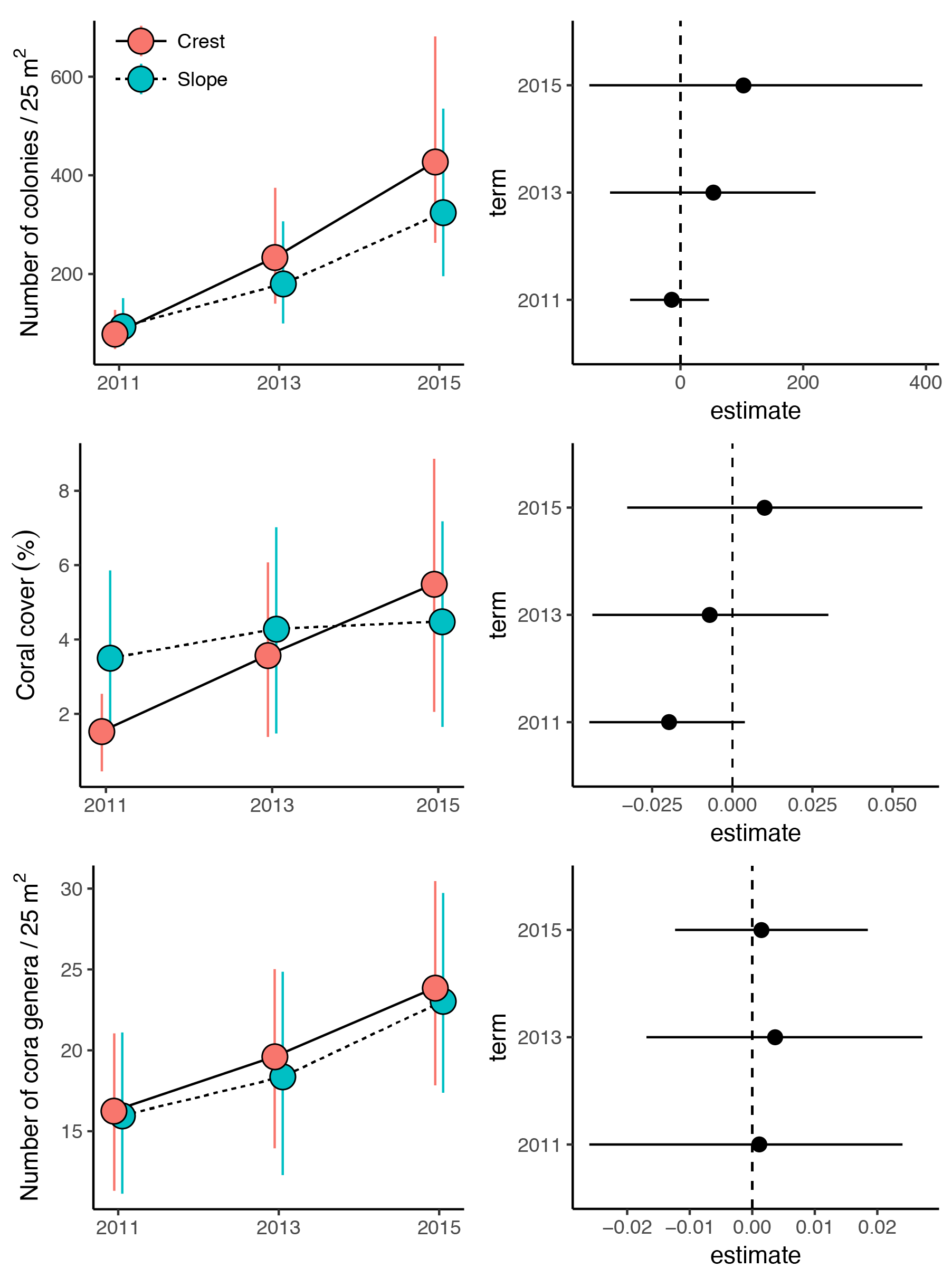


**Fig. S3 .** Modeled mean +- 95% credibility interval of colony density (a), coral cover (b), and genus diversity (c) by depth and year on the exposed fringing reef of Orpheus Island, central GBR. Data obtained from three replicate 5 x 5 m quadrats per depth zone per time-point (bar the slope in 2013, when only two quadrats were surveyed for logistical constraints). The Bayesian generalized linear mixed effects models used negative binomial (a), binomial (b), and poisson (c) distributions, respectively. Corresponding caterpillar-plots indicate the 95% credibility intervals of comparisons between the crest and slope data at each time-point, where zero represents no difference.


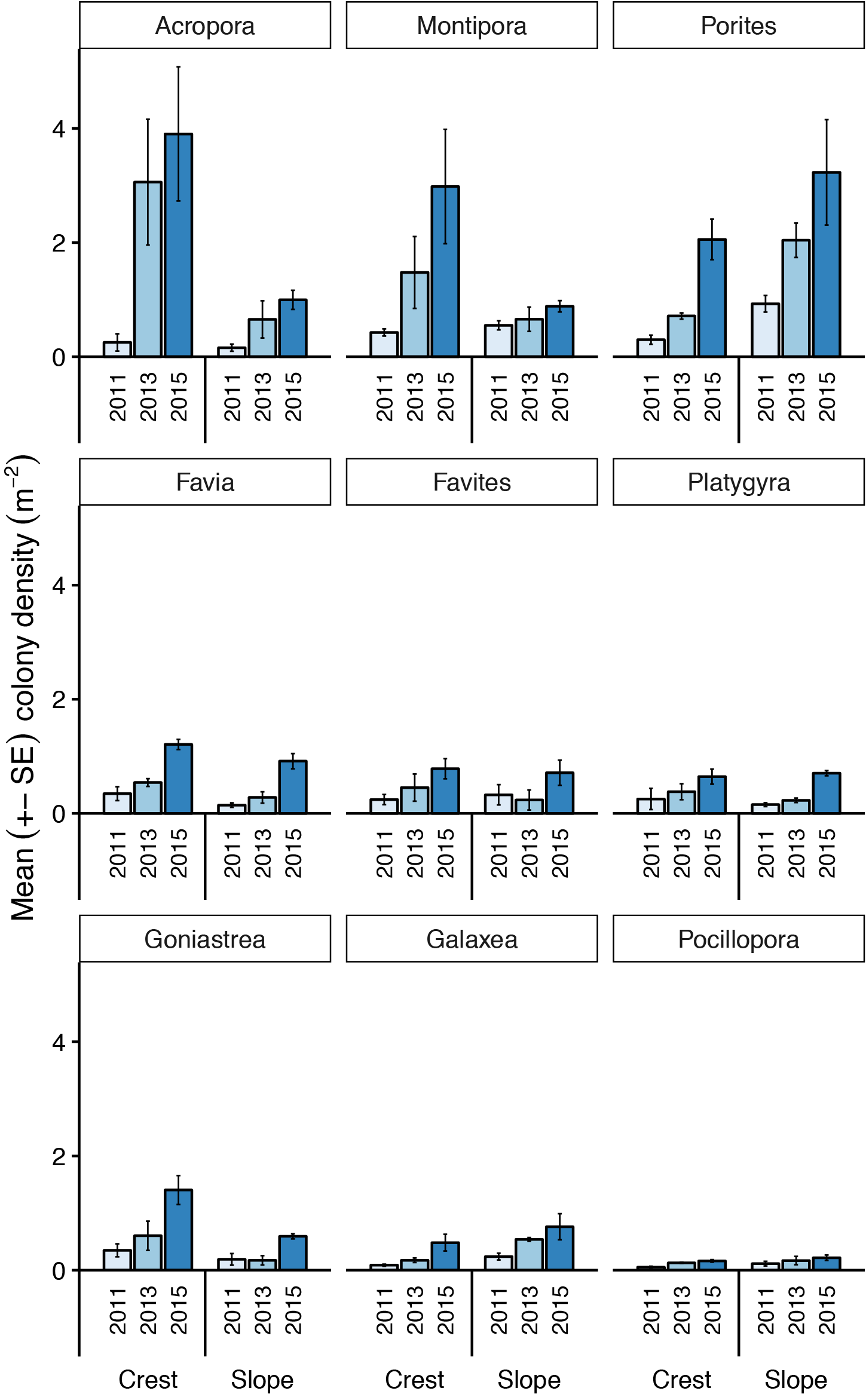


**Fig. S4** Mean (± SE) density of the most common coral genera (per m^2^) by depth and year at E Orpheus, central GBR. All colonies ≥1 cm mean diameter. n=3 quadrats per zone, except in 2013 when only two quadrats were surveyed on the lower slope.


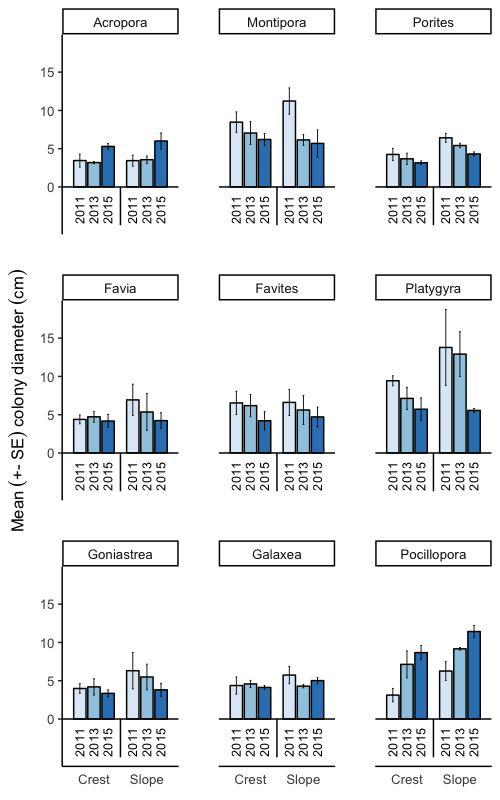


**Fig. S5** Mean (± SE) colony area of the most common coral genera by depth and year at E Orpheus, central GBR. All colonies ≥1 cm mean diameter. n=3 quadrats per zone, except in 2013 when only two quadrats were surveyed on the lower slope.

**Table S1.** Re-classification of taxonomically resolved data from post-2008 into the broad morphological groups of Gralton^46^; following Veron^47^.

| **Branching/Arborescent** |
| --- |
| Acropora: bushy, branching, staghorn, columnar; Hydnophora, Isopora, Tubastrea |
|  |
| **Corymbose/Plating** |
| Acropora: bottlebrush, clumping, digitate, tabular, corymbose; Pocilloporidae; Turbinaria; Pachyseris; Merulina; Echinopora; Leptoseris; Pavona |
|  |
| **Encrusting** |
| Montipora; Cyphastrea; Leptastrea; Galaxea; Echinophyllia; Mycedium; Scolymia; Trachiphyllia; Oxypora; Trachyphyllia |
|  |
| **Massive** |
| Acanthastrea; Alveopora; Astreopora; Caulastrea; Coeloseris; Diploastrea; Favia; Favites; Goniastrea; Goniopora; Leptoria; Lobophyllia; Montastrea; Oulophyllia; Pectinia; Platygyra; Plerogyra; Symphyllia; Physogyra; Gardineroseris; Psammocora; Coscinarea |

**Table S2.** List of R packages and functions used for data analysis.

**data manipulation and graphing**

tidyverse

ggplot2

**Bayesian analyses**

rstanarm::: stan_glmer

broom::: tidyMCMC

multcomp::: contrMat

**nMDS**

vegan:::metaMDS

**Pairwise permutational MANOVA**

RVAideMemoire:::pairwise.perm.manova

**Table S3**. Bayesian models and their parameter estimates. ‘rhat’ vales are a measure of the efficiency of the model. Values under 1.05 indicate that the model was appropriately parameterised.

A) Changes in overall coral cover on transects

coral.stanT = stan_glmer(cbind(Cover, 2000-Cover)~Year +(1|Site),

data=coral,

family='binomial',

prior = normal(1, 10),

prior_intercept = normal(0,10),

chains = 3,

iter = 3000, thin=4, warmup=1000)

| **term** | **estimate** | **std.error** | **cred.low** | **cred.high** |
| --- | --- | --- | --- | --- |
| (Intercept) | -0.94856112 | 0.4082642 | -1.922812678 | -0.10053666 |
| Year2011 | -2.20535691 | 0.07220537 | -2.346679446 | -2.06186103 |
| Year2014 | -3.16564342 | 0.10602803 | -3.367243835 | -2.97421398 |
| Year2017 | -0.03860928 | 0.04046906 | -0.115015432 | 0.04117919 |
| b[(Intercept) Site:EP] | 0.10641447 | 0.40641207 | -0.744402644 | 1.07174884 |
| b[(Intercept) Site:NEO] | -0.12518173 | 0.40784124 | -0.99404826 | 0.81902606 |
| Sigma[Site:(Intercept),(Intercept)] | 0.44405262 | 0.96773289 | 0.002350304 | 1.91124043 |

B) Changes in coral abundance among years and reef zones

dens.stan1 <- stan_glmer(CC~Year*Depth + (1|Quadrat), data=dens,

family='neg_binomial_2',

prior=normal(0,1),

prior_intercept=normal(0,10),

prior_aux=cauchy(0,5),

chains=3, iter=5000, warmup=2000, thin=4)

| **term** | **estimate** | **std.error** | **conf.low** | **conf.high** | **rhat** |
| --- | --- | --- | --- | --- | --- |
| (Intercept) | 4.35E+00 | 0.2542796 | 3.85E+00 | 4.8527152 | 1.0026229 |
| Year2013 | 1.07E+00 | 0.2933655 | 5.33E-01 | 1.665035 | 0.9997379 |
| Year2015 | 1.67E+00 | 0.2872341 | 1.05E+00 | 2.2145798 | 1.0000198 |
| DepthSlope | 1.43E-01 | 0.3638069 | -6.55E-01 | 0.8480183 | 1.0000571 |
| Year2013:DepthSlope | -4.13E-01 | 0.4418997 | -1.24E+00 | 0.5119869 | 0.9990064 |
| Year2015:DepthSlope | -4.10E-01 | 0.3917385 | -1.18E+00 | 0.3893049 | 1.0000369 |
| b[(Intercept) Quadrat:1] | -5.31E-02 | 0.1759101 | -4.51E-01 | 0.2581093 | 1.0012044 |
| b[(Intercept) Quadrat:2] | 7.73E-02 | 0.1894509 | -2.13E-01 | 0.5148563 | 1.0025465 |
| b[(Intercept) Quadrat:3] | -1.10E-02 | 0.1716177 | -3.65E-01 | 0.3457381 | 0.9997093 |
| b[(Intercept) Quadrat:4] | 1.18E-02 | 0.1769059 | -3.54E-01 | 0.3972622 | 0.9993536 |
| b[(Intercept) Quadrat:6] | 3.43E-02 | 0.1907699 | -2.88E-01 | 0.4763067 | 1.0003544 |
| b[(Intercept) Quadrat:9] | -2.05E-02 | 0.1784199 | -4.43E-01 | 0.314905 | 0.9987874 |
| b[(Intercept) Quadrat:_NEW_Quadrat] | 4.91E-03 | 0.2539132 | -4.64E-01 | 0.4908148 | 1.001211 |
| reciprocal_dispersion | 1.15E+01 | 6.2110491 | 2.61E+00 | 24.0338409 | 1.0006269 |
| Sigma[Quadrat:(Intercept),(Intercept)] | 6.92E-02 | 0.259267 | 2.24E-09 | 0.2640563 | 1.0017004 |
| mean_PPD | 2.25E+02 | 34.5886975 | 1.69E+02 | 291.5294118 | 1.0002843 |
| log-posterior | -1.16E+02 | 3.514065 | -1.23E+02 | -109.6424875 | 0.999666 |

C) Changes in coral cover among years and reef zones

cover.stan = stan_glmer(cbind(CC, 1000-CC)~Year*Depth +(1|Quadrat),

data=cover,

family='binomial',

prior = normal(0, 10),

prior_intercept = normal(0,10),

chains = 3,

iter = 3000, thin=4, warmup=1000)

| **term** | **estimate** | **std.error** | **conf.low** | **conf.high** | **rhat** |
| --- | --- | --- | --- | --- | --- |
| (Intercept) | -4.253653148 | 0.3504178 | -4.94732088 | -3.5317404 | 1.0028663 |
| Year2013 | 0.885273661 | 0.1806585 | 0.54277565 | 1.259852 | 1.0020156 |
| Year2015 | 1.335273718 | 0.1713245 | 1.01380649 | 1.6756316 | 1.0024984 |
| DepthSlope | 0.873523098 | 0.497188 | -0.01628691 | 1.8942532 | 1.0004419 |
| Year2013:DepthSlope | -0.686107496 | 0.2489251 | -1.19797363 | -0.2588738 | 1.0037702 |
| Year2015:DepthSlope | -1.075006261 | 0.2146682 | -1.48558823 | -0.6638505 | 1.0061448 |
| b[(Intercept) Quadrat:1] | 0.078678448 | 0.3278945 | -0.62432573 | 0.7284098 | 1.0007688 |
| b[(Intercept) Quadrat:2] | 0.259704181 | 0.3311325 | -0.47547221 | 0.8964124 | 1.0012467 |
| b[(Intercept) Quadrat:3] | -0.305878599 | 0.3270715 | -0.89268127 | 0.4324411 | 1.0001768 |
| b[(Intercept) Quadrat:4] | 0.016394632 | 0.3377735 | -0.68049439 | 0.6912069 | 1.0004737 |
| b[(Intercept) Quadrat:6] | 0.448051355 | 0.3447756 | -0.19358245 | 1.1958758 | 1.0007091 |
| b[(Intercept) Quadrat:9] | -0.445433133 | 0.337766 | -1.1676513 | 0.1669979 | 0.9996634 |
| b[(Intercept) Quadrat:_NEW_Quadrat] | -0.006251365 | 0.5651388 | -1.03546093 | 1.241419 | 1.0019836 |
| Sigma[Quadrat:(Intercept),(Intercept)] | 0.317864507 | 0.367289 | 0.02633018 | 0.9392281 | 0.9988992 |
| mean_PPD | 36.32537255 | 2.0180732 | 32.52941176 | 40.2941176 | 1.0013888 |
| log-posterior | -80.73876931 | 2.9715087 | -86.42252669 | -75.4395687 | 0.9988674 |

D) Changes in coral genus diversity among years and reef zones

div.stan2 <- stan_glmer(CDr~Year*Depth + (1|Quadrat), data=div,

family='poisson',

prior=normal(0,1),

prior_intercept=normal(0,10),

#prior_aux=cauchy(0,5),

chains=3, iter=6000, warmup=3000, thin=4)

| **term** | **estimate** | **std.error** | **conf.low** | **conf.high** | **rhat** |
| --- | --- | --- | --- | --- | --- |
| (Intercept) | 2.77E+00 | 0.15531502 | 2.46E+00 | 3.06136854 | 1.0003544 |
| Year2013 | 1.92E-01 | 0.17999236 | -1.80E-01 | 0.52301218 | 1.0001002 |
| Year2015 | 3.95E-01 | 0.17568832 | 4.70E-02 | 0.74393642 | 0.9992088 |
| DepthSlope | -7.63E-03 | 0.21859844 | -4.33E-01 | 0.41201869 | 1.0018669 |
| Year2013:DepthSlope | -5.54E-02 | 0.2748104 | -6.07E-01 | 0.48469759 | 1.002348 |
| Year2015:DepthSlope | -3.32E-02 | 0.24881388 | -5.23E-01 | 0.45165847 | 1.0003331 |
| b[(Intercept) Quadrat:1] | 4.57E-02 | 0.11041394 | -1.54E-01 | 0.27577874 | 1.0005919 |
| b[(Intercept) Quadrat:2] | -3.21E-02 | 0.10422715 | -2.79E-01 | 0.14930766 | 1.0000252 |
| b[(Intercept) Quadrat:3] | -8.59E-03 | 0.10177788 | -2.29E-01 | 0.19054738 | 1.0004375 |
| b[(Intercept) Quadrat:4] | 2.10E-02 | 0.10371422 | -1.70E-01 | 0.25853089 | 0.9991245 |
| b[(Intercept) Quadrat:6] | -3.61E-03 | 0.10466807 | -2.26E-01 | 0.2152257 | 1.0004727 |
| b[(Intercept) Quadrat:9] | -1.11E-02 | 0.10097461 | -2.39E-01 | 0.20233279 | 0.9998637 |
| b[(Intercept) Quadrat:_NEW_Quadrat] | 5.26E-04 | 0.14040876 | -2.94E-01 | 0.29270519 | 1.0013108 |
| Sigma[Quadrat:(Intercept),(Intercept)] | 2.10E-02 | 0.06352247 | 4.01E-10 | 0.08072529 | 1.0006273 |
| mean_PPD | 1.96E+01 | 1.48946599 | 1.65E+01 | 22.35294118 | 1.0010502 |
| log-posterior | -6.61E+01 | 2.9062787 | -7.15E+01 | -60.77491693 | 0.9999412 |

**Table S4.** Modeled mean density, coral cover and genus diversity on crest and slope at exposed reefs of Orpheus Island.

| **Colony density** | |  |  |  |
| --- | --- | --- | --- | --- |
| **Year** | **Zone** | **Mean density** | **95% credibility interval** | **Probability** |
| 2011 | Crest | 79.02919 | 47.96-127.1 | P_crest>slope_ = 0.29 |
| 2013 | Crest | 234.04376 | 139.79-374.41 | P_crest>slope_ = 0.79 |
| 2015 | Crest | 427.16075 | 263.31-681.38 | P_crest>slope_ = 0.81 |
| 2011 | Slope | 93.57426 | 55.72-150.97 | P_slope>crest_ = 0.71 |
| 2013 | Slope | 179.91308 | 99.83-306.58 | P_slope>crest_ = 0.21 |
| 2015 | Slope | 324.79258 | 195.39-535.08 | P_slope>crest_ = 0.19 |
|  |  |  |  |  |
| **Coral cover** | |  |  |  |
| **Year** | **Zone** | **Mean cover** | **95% credibility interval** | **Probability** |
| 2011 | Crest | 1.529514 | 0.45-2.54 | P_crest>slope_ = 0.04 |
| 2013 | Crest | 3.579925 | 1.38-6.07 | P_crest>slope_ = 0.32 |
| 2015 | Crest | 5.495453 | 2.05-8.86 | P_crest>slope_ = 0.71 |
| 2011 | Slope | 3.498884 | 1.62-5.86 | P_slope>crest_ = 0.96 |
| 2013 | Slope | 4.286865 | 1.47-7.02 | P_slope>crest_ = 0.68 |
| 2015 | Slope | 4.48815 | 1.65-7.18 | P_slope>crest_ = 0.29 |
|  |  |  |  |  |
| **Genus diversity** | |  |  |  |
| **Year** | **Zone** | **Mean # of genera** | **95% credibility interval** | **Probability** |
| 2011 | Crest | 2.505468 | 11.32-21.05 | P_crest>slope_ = 0.53 |
| 2013 | Crest | 2.864307 | 13.95-25.02 | P_crest>slope_ = 0.62 |
| 2015 | Crest | 3.25602 | 17.84-30.46 | P_crest>slope_ = 0.57 |
| 2011 | Slope | 2.597438 | 11.15-21.1 | P_slope>crest_ = 0.47 |
| 2013 | Slope | 3.233413 | 12.29-24.85 | P_slope>crest_ = 0.38 |
| 2015 | Slope | 3.198327 | 17.38-29.73 | P_slope>crest_ = 0.43 |

**Table S5.** Names of data contributors to the study and details of data supplied

| Name | Initials | Year | Data collection method | Details |
| --- | --- | --- | --- | --- |
| Bette Willis | BW | 1998  1999  2001 | Line intercept transects | Coral composition / cover |
| Cathie Page | CP | 1999 | Line intercept transects | Coral composition / cover |
| Charlotte Gralton | CG | 2001 | Line intercept transects | Coral composition / cover |
| Yui Sato | YS | 2008 | Line intercept transects | Coral composition / cover |
| David Bourne | DB | 2008 | Line intercept transects | Coral composition / cover |
| Vimoksalehi Lukoschek | VL | 2011 | Line intercept transects  Belt transects | Coral composition / benthic cover |
| Peter Cross | PC | 2011 | Permanent quadrats | Taxonomic identity, abundance and colony size |
| Gergely Torda | GT | 2011  2013  2015  2014, 2017 | Permanent quadrats  Line intercept transects  Belt transects | Taxonomic identity, abundance and colony size, structural complexity  Coral composition / benthic cover |
| Katie Sambrook | KS | 2015  2014 | Permanent quadrats  Line intercept transects  Belt transects | Taxonomic identity, abundance and colony size  Coral composition / benthic cover |
|  |  |  |  |  |

**R Scripts**

library(rstanarm)

library(rstan)

library(broom)

library(tidyverse)

library(ggplot2)

library(gridExtra)

library(ggfortify)

library(sjPlot)

library(MuMIn)

library(stringr)

df <- read.csv('LIT.csv', strip.white = T)

SE <- function(x) sd(x)/sqrt(length(x))

##Graphs

### Stacked bar graph

coralcover <- df %>%

filter(Category != 'Soft corals') %>%

unite(SiTr, c(Site, Transect), sep = '-') %>%

group_by(Year, SiTr, Category) %>%

summarise(percent.cover=sum(LIT)/20) %>%

group_by(Year, SiTr) %>%

mutate(Cov.Tot = sum(percent.cover), Cov.Prop = 100*percent.cover/Cov.Tot) %>%

group_by(Year, Category) %>%

summarise(Cov.Mean=mean(percent.cover), Cov.SE=SE(percent.cover),

Cov.Prop.Mean = mean(Cov.Prop), Cov.Prop.SE=SE(Cov.Prop))

coralcover$Category <- factor(coralcover$Category, levels = c("Acropora", "Montipora", "Pocilloporidae", "Faviidae", "Poritidae", "Other HC"))

ggplot(coralcover, aes(y=Cov.Mean, x=Year, fill=Category, color = Category)) +

geom_bar(stat='identity', position = 'stack', alpha = 0.2, width = 1) +

theme_classic(12) +

theme(axis.text.x = element_text(angle = 45, hjust = 1)) +

theme(axis.line.x = element_line(color='black', size=0.5)) +

theme(axis.line.y = element_line(color='black', size=0.5)) +

scale_y_continuous(expression(Mean~'%'~coral~cover)) +

theme(legend.position='right') +

scale_x_continuous(expression(), breaks=c(2008, 2011, 2014, 2017)) +

theme(legend.title=element_blank()) +

theme(strip.text.x = element_blank()) +

annotate("segment", x=-Inf, xend=Inf, y=-Inf, yend=-Inf, size = 1)+

annotate("segment", x=-Inf, xend=-Inf, y=-Inf, yend=Inf, size = 1) +

theme(panel.margin = unit(2, "lines"))

### Pointrange graphs per Genus

coralcover2 <- df %>%

filter(Category != 'Soft corals') %>%

group_by(Year, Site, Transect, Category) %>%

summarise(percent.cover=sum(LIT)/20) %>%

group_by(Year, Site, Transect) %>%

mutate(Cov.Tot = sum(percent.cover), Cov.Prop = 100*percent.cover/Cov.Tot) %>%

group_by(Year, Site, Category) %>%

summarise(Cov.Mean=mean(percent.cover), Cov.SE=SE(percent.cover),

Cov.Prop.Mean = mean(Cov.Prop), Cov.Prop.SE=SE(Cov.Prop))

coralcover2$Category <- factor(coralcover2$Category, levels = c("Acropora", "Montipora", "Pocilloporidae", "Faviidae", "Poritidae", "Other HC"))

ggplot(coralcover2, aes(y=Cov.Mean, x=Year, color = Category, shape = Site)) +

geom_pointrange(aes(ymax = Cov.Mean + Cov.SE, ymin = Cov.Mean - Cov.SE),

size=0.5, position = position_dodge(1.1)) +

geom_line(aes(linetype = Site), alpha = 0.5, position = position_dodge(1.1))+

facet_wrap(~Category, nrow = 2, ncol = 3, scales = 'free') +

theme_classic(12) +

theme(axis.text.x = element_text(angle = 45, hjust = 1)) +

theme(axis.line.x = element_line(color='black', size=0.1)) +

theme(axis.line.y = element_line(color='black', size=0.1)) +

scale_y_continuous(expression(Mean~'%'~coral~cover)) +

theme(legend.position='right') +

scale_x_continuous(expression(), breaks=c(2008, 2011, 2014, 2017)) +

theme(legend.title=element_blank()) +

scale_shape_manual(values = c(15,16)) +

scale_linetype_manual(values = c('dashed', 'dotted'))

###historical outlook, stacked bars

historical <- read.csv('historical.csv', header=T, strip.white=T)

df$Morphology[df$Sp %in% c('bushy', 'branching', 'staghorn', 'columnar')] <- 'Branching/Arborescent'

df$Morphology[df$Genus %in% c('Hydnophora', 'Isopora', 'Tubastrea')] <- 'Branching/Arborescent'

df$Morphology[df$Family %in% c('Pocilloporidae')] <- 'Corymbose/Plating'

df$Morphology[df$Sp %in% c('bottlebrush', 'clumping', 'digitate', 'tabular', 'corymbose')] <- 'Corymbose/Plating'

df$Morphology[df$Genus %in% c('Turbinaria', 'Pachyseris', 'Merulina', 'Echinopora', 'Leptoseris', 'Pavona')] <- 'Corymbose/Plating'

df$Morphology[df$Sp %in% c('encrusting')] <- 'Encrusting'

df$Morphology[df$Genus %in% c('Montipora', 'Cyphastrea', 'Leptastrea', 'Galaxea', 'Echinophyllia', 'Mycedium', 'Scolymia', 'Trachiphyllia', 'Oxypora', 'Styloco/Palaus/Madrac', 'Trachyphyllia')] <- 'Encrusting'

df$Morphology[df$Genus %in% c('Acanthastrea', 'Alveopora', 'Astreopora', 'Caulastrea', 'Coeloseris', 'Diploastrea', 'Favia', 'Favites', 'Goniastrea', 'Goniopora', 'Leptoria', 'Lobophyllia', 'Montastrea', 'Oulophyllia', 'Pectinia', 'Platygyra', 'Plerogyra', 'Faviidae', 'Symphyllia', 'Physogyra', 'Gardineroseris','Psammocora','Coscinarea')] <- 'Massive'

df$Morphology[df$Sp %in% c('massive')] <- 'Massive'

coralcover3 <- df %>%

filter(Category != 'Soft corals') %>%

filter(Morphology != 'NA') %>%

unite(SiTr, c(Site, Transect), sep = '-') %>%

group_by(Year, SiTr, Morphology) %>%

summarise(percent.cover=sum(LIT)/20) %>%

group_by(Year, SiTr) %>%

mutate(Cov.Tot = sum(percent.cover), Cov.Prop = 100*percent.cover/Cov.Tot) %>%

group_by(Year, Morphology) %>%

summarise(Cov.Mean=mean(percent.cover), Cov.SE=SE(percent.cover)) %>%

as.data.frame()

final <-rbind(historical, coralcover3)

final$Morphology <- as.factor(final$Morphology)

final$Morphology <- factor(final$Morphology, levels = rev(c("Branching/Arborescent", "Corymbose/Plating", "Encrusting", "Massive")))

ggplot(final, aes(y=Cov.Mean, x=Year, fill=Morphology)) +

geom_bar(stat='identity', position = 'stack', alpha = 0.2, color = 'gray') +

theme_classic(12) +

theme(axis.text.x = element_text(angle = 45, hjust = 1)) +

theme(axis.line.x = element_line(color='black', size=0.5)) +

theme(axis.line.y = element_line(color='black', size=0.5)) +

scale_y_continuous(expression(Mean~'%'~coral~cover)) +

#theme(legend.position='bottom') +

theme(legend.position = c(1,1), legend.justification = c(1,1)) +

scale_x_continuous(expression(), breaks=c(1998, 1999, 2001, 2008, 2011, 2014, 2017)) +

theme(legend.title=element_blank()) +

#scale_color_brewer(palette = 'Greys') +

scale_fill_brewer(palette = 'Greys') +

guides(colour = guide_legend(reverse=T), fill= guide_legend(reverse=T))

###historical outlook, pointrange graphs per morphology

final$Morphology <- factor(final$Morphology, levels = c("Branching/Arborescent", "Corymbose/Plating", "Encrusting", "Massive"))

ggplot(final, aes(y=Cov.Mean, x=Year)) +

geom_pointrange(aes(ymax = Cov.Mean + Cov.SE, ymin = Cov.Mean - Cov.SE), size=0.3) +

geom_line(alpha = 0.3, linetype = 'dotted')+

facet_wrap(~Morphology, nrow = 4, ncol = 2, scales = 'free') +

theme_classic(12) +

theme(axis.text.x = element_text(angle = 45, hjust = 1)) +

theme(axis.line.x = element_line(color='black', size=0.5)) +

theme(axis.line.y = element_line(color='black', size=0.5)) +

scale_y_continuous(expression(Mean~'%'~coral~cover)) +

theme(legend.position='bottom') +

scale_x_continuous(expression(), breaks=c(1998, 1999, 2001, 2008, 2011, 2014, 2017)) +

theme(legend.title=element_blank()) +

theme(strip.background = element_rect(fill="#CCCCFF"))

#### some basic descriptive stats for the paper

#stats for paper

dstat <- final %>%

group_by(Year) %>%

summarise(total = sum(Cov.Mean))

#decrease:

1-dstat[7,'total']/dstat[1,'total']

#recovery rate:

(dstat[4,'total']-dstat[2,'total'])/(dstat[4,'Year']-dstat[2,'Year'])

(dstat[7,'total']-dstat[6,'total'])/(dstat[7,'Year']-dstat[6,'Year'])

(dstat[3,'total']-dstat[2,'total'])/(dstat[3,'Year']-dstat[2,'Year'])

(dstat[4,'total']-dstat[3,'total'])/(dstat[4,'Year']-dstat[3,'Year'])

#descriptive stats decreases by morphology

mstat <- final %>%

group_by(Year, Morphology) %>%

summarise(total = sum(Cov.Mean))

1-mstat[5,'total']/mstat[1,'total']

1-mstat[6,'total']/mstat[2,'total']

1-mstat[7,'total']/mstat[3,'total']

1-mstat[8,'total']/mstat[4,'total']

1-mstat[17,'total']/mstat[13,'total']

1-mstat[18,'total']/mstat[14,'total']

1-mstat[19,'total']/mstat[15,'total']

1-mstat[20,'total']/mstat[16,'total']

1-mstat[13,'total']/mstat[9,'total']

1-mstat[16,'total']/mstat[12,'total']

dom <- mstat %>%

group_by(Year) %>%

summarise(dom = max(total)/sum(total))

#decreases by category after Yasi

CatStat <- df %>%

filter(Morphology != 'NA') %>%

unite(SiTr, c(Site, Transect), sep = '-') %>%

group_by(Year, SiTr, Category) %>%

summarise(percent.cover=sum(LIT)/20)

###NMDS plot and associated stats

library(vegan)

nmds <- df %>%

filter(Category != 'Other HC') %>%

mutate(Cat = factor(ifelse(Genus == 'Acropora', paste0(Genus, ' (', Sp, ')'), as.character(Genus)))) %>%

dplyr:::select(Year, Site, Transect, Cat, LIT) %>%

group_by(Year, Site, Transect, Cat) %>%

summarise(sumLIT = sum(LIT)) %>%

spread(Cat, sumLIT)

nmds[is.na(nmds)] <- 0

nmds.stnd <- nmds %>% ungroup() %>%

mutate(Transect = as.factor(Transect)) %>%

mutate_if(is.numeric, funs(decostand(.^0.25, MARGIN = 2, 'max')))

franko.mds <- metaMDS(nmds.stnd[, c(-1:-3)], k = 2, autotransform = FALSE)

#stressplot(franko.mds)

#plot(franko.mds)

franko.site.scores <- as.data.frame(scores(franko.mds, display = 'sites'))

franko.site.scores <- data.frame(franko.site.scores, nmds) #so that we have the original names

franko.species.scores <- as.data.frame(scores(franko.mds, display = 'species'))

franko.species.scores$Species <- rownames(franko.species.scores)

#head(franko.species.scores)

#head(franko.site.scores)

franko.site.scores$Year <- as.factor(franko.site.scores$Year)

hjust <- ifelse(franko.species.scores$NMDS1>0, 0, 1)

vjust <- ifelse(franko.species.scores$NMDS2>0, 0, 1)

g <- ggplot() +

geom_segment(data = NULL, aes(y=-Inf, x = 0, yend = Inf, xend = 0), linetype = 'dotted') +

geom_segment(data = NULL, aes(y=0, x = -Inf, yend = 0, xend = Inf), linetype = 'dotted') +

geom_point(data = franko.site.scores, aes(y = NMDS2, x = NMDS1, color = Year), size = 2) +

geom_segment(data = franko.species.scores, aes(y = 0, x = 0, yend = NMDS2, xend = NMDS1), arrow = arrow(length = unit(0.3, 'lines')), alpha = 0.2) +

geom_text(data = franko.species.scores, aes(y = NMDS2, x = NMDS1, label = Species, hjust = hjust, vjust = vjust), show.legend = F, size = 3, color = 'gray') +

theme_classic() +

scale_y_continuous(expression()) +

scale_x_continuous(expression()) +

coord_equal(xlim = c(-1.3, 1.5), ylim = c(-1, 1)) +

theme(legend.position = c(0.15, 1), legend.justification = c(1,1))

g

franko.hull <- franko.site.scores %>% group_by(Year) %>%

do({

x = .

x[chull(x$NMDS1, x$NMDS2),] #function chull creates a convex hull for xy coordinates

})

g <- g + geom_polygon(data = franko.hull, aes(y=NMDS2, x=NMDS1, fill = Year), alpha = 0.2)

g

#ggsave('nmds.pdf', g, width = 7, height = 7, units = 'in')

#simper

ComMat <- nmds %>% ungroup() %>% dplyr:::select(-1:-3)

nmds <- nmds %>% ungroup() %>% mutate(Year = as.factor(Year))

nmds.sim <- simper(ComMat, nmds$Year)

summary(nmds.sim)

#permanova

library(RVAideMemoire)

nmds.dist <- vegdist(nmds.stnd[, -1:-3], 'bray')

pairwise.perm.manova(nmds.dist, nmds$Year)

##Bayesian models

sp <- 'Acropora'

coral <- df %>%

filter(Category == sp) %>%

#unite(SiTr, c(Site, Transect), sep = '-') %>%

group_by(Year, Site, Transect, Category) %>%

summarise(Cover=sum(LIT)) %>% #the 'number of 'success' for each taxon out of 2000 'trials'

ungroup() %>%

mutate(Year = as.factor(Year), Transect = as.factor(Transect))

coral

levels(coral$Year)

ggplot(coral, aes(Year, Cover/20, fill = Site)) + geom_boxplot() #divide by 20 to get the % cover on 20 m

coral <- coral %>%

group_by(Year, Site) %>%

mutate(dropit = SE(Cover) == 0) %>%

filter(dropit != 'TRUE') %>%

droplevels()

table(coral$Cover ==0)

levels(coral$Year)

#leave out no-variance timepoints, as stats cannot be calculated on no vairance. use % instead of proportions, cause bayesian bionmial won't run on non-integers. Keep sites as random effects, otherwise its pseudo-repliaction (transects don't represent the reef, they represent the sites, and the sites represent the reef).

#bayesian:

coral.stanA = stan_glmer(cbind(Cover, 2000-Cover)~Year +(1|Site), #Cover is success, 2000-Cover is failure.

data=coral,

family='binomial',

prior = normal(1, 10),

prior_intercept = normal(0,10),

chains = 3,

iter = 6000, thin=6, warmup=2000)

stan_trace(coral.stanA)

stan_ac(coral.stanA)

posterior_vs_prior(coral.stanA, group_by_parameter = TRUE, facet_args = list(scales = "free_y"),prob = .95)

tidyMCMC(coral.stanA, conf.int = T, conf.method = 'HPDinterval')

newdata = with(coral, expand.grid(Year = levels(Year)))

Xmat = model.matrix(~Year, newdata)

coefs = as.data.frame(coral.stanA) %>% dplyr:::select(starts_with('(Intercept)'), starts_with('Year')) %>% as.matrix()

fit = binomial()$linkinv(coefs %*% t(Xmat))

newdata = newdata %>% cbind(tidyMCMC(fit, conf.int = T, conf.method = 'HPDinterval')) %>%

mutate_if(is.numeric, funs(.*100)) %>%

mutate(Year = as.numeric(as.character(Year)))

summary(newdata)

(assign(paste0(sp, 'Plot'), ggplot(newdata, aes(y=estimate, x=Year)) +

geom_linerange(aes(ymin = conf.low, ymax = conf.high)) +

geom_point() +

theme_classic()+

scale_y_continuous('Cover (%)') +

scale_x_continuous('', breaks = c(2008, 2011, 2014, 2017)) +

ggtitle(sp)

))

#Tukey's comparisons

library(multcomp)

library(coda)

newdata = with(coral, expand.grid(Year = levels(Year)))

Xmat = model.matrix(~Year, newdata)

coefs = as.data.frame(coral.stanA) %>% dplyr:::select(starts_with('(Intercept)'), starts_with('Year')) %>% as.matrix()

fit = binomial()$linkinv(coefs %*% t(Xmat))

tuk.mat <- contrMat(n=table(newdata$Year), type='Tukey')

fit = fit %*% t(tuk.mat)

head(fit)

colMeans(fit)

comp <- tidyMCMC(as.mcmc(fit), conf.int=TRUE,conf.method='HPDinterval')

(AcroporaButterfly <- ggplot(comp, aes(y=estimate, x=term)) +

geom_pointrange(aes(ymin=conf.low, ymax=conf.high)) +

geom_hline(yintercept=0, linetype='dashed') +

#scale_x_continuous(breaks = c(0.5, 1, 1.5))

coord_flip() +

theme_classic() +

ggtitle(''))

#Specific questions:

newdata = with(coral, expand.grid(Year = levels(Year)))

Xmat = model.matrix(~Year, newdata)

coefs = as.data.frame(coral.stanA) %>% dplyr:::select(starts_with('(Intercept)'), starts_with('Year')) %>% as.matrix()

fit = binomial()$linkinv(coefs %*% t(Xmat))

newdata = newdata %>% cbind(tidyMCMC(fit, conf.int = T, conf.method = 'HPDinterval'))

head(newdata)

colnames(fit)

Diff.abs <- fit[, 3] - fit[,1]

sum(Diff.abs>0)/length(Diff.abs)

tidyMCMC(as.mcmc(Diff.abs), conf.int = T, conf.method = 'HPDinterval')

Diff.perc <- (fit[,3]-fit[,1])/fit[,1]

tidyMCMC(as.mcmc(Diff.perc), conf.int = T, conf.method = 'HPDinterval')

colMeans(fit)

##################

sp <- 'Montipora'

coral <- df %>%

filter(Category == sp) %>%

#unite(SiTr, c(Site, Transect), sep = '-') %>%

group_by(Year, Site, Transect, Category) %>%

summarise(Cover=sum(LIT)) %>%

ungroup() %>%

mutate(Year = as.factor(Year), Transect = as.factor(Transect))

coral

levels(coral$Year)

ggplot(coral, aes(Year, Cover/20, fill = Site)) + geom_boxplot()

coral <- coral %>%

group_by(Year, Site) %>%

mutate(dropit = SE(Cover) == 0) %>%

filter(dropit != 'TRUE') %>%

droplevels()

table(coral$Cover ==0)

levels(coral$Year)

coral.stanM = stan_glmer(cbind(Cover, 2000-Cover)~Year +(1|Site),

data=coral,

family='binomial',

prior = normal(1, 10),

prior_intercept = normal(0,10),

chains = 3,

iter = 3000, thin=4, warmup=1000)

stan_trace(coral.stanM)

stan_ac(coral.stanM)

posterior_vs_prior(coral.stanM, group_by_parameter = TRUE, facet_args = list(scales = "free_y"),prob = .95)

tidyMCMC(coral.stanM, conf.int = T, conf.method = 'HPDinterval')

newdata = with(coral, expand.grid(Year = levels(Year)))

Xmat = model.matrix(~Year, newdata)

coefs = as.data.frame(coral.stanM) %>% dplyr:::select(starts_with('(Intercept)'), starts_with('Year')) %>% as.matrix()

fit = binomial()$linkinv(coefs %*% t(Xmat))

newdata = newdata %>% cbind(tidyMCMC(fit, conf.int = T, conf.method = 'HPDinterval')) %>%

mutate_if(is.numeric, funs(.*100)) %>%

mutate(Year = as.numeric(as.character(Year)))

summary(newdata)

(assign(paste0(sp, 'Plot'), ggplot(newdata, aes(y=estimate, x=Year)) +

geom_linerange(aes(ymin = conf.low, ymax = conf.high)) +

geom_point() +

theme_classic()+

scale_y_continuous('Cover (%)') +

scale_x_continuous('', breaks = c(2008, 2011, 2014, 2017)) +

ggtitle(sp)

))

#Tukey's comparisons

newdata = with(coral, expand.grid(Year = levels(Year)))

Xmat = model.matrix(~Year, newdata)

coefs = as.data.frame(coral.stanM) %>% dplyr:::select(starts_with('(Intercept)'), starts_with('Year')) %>% as.matrix()

fit = binomial()$linkinv(coefs %*% t(Xmat))

tuk.mat <- contrMat(n=table(newdata$Year), type='Tukey')

fit = fit %*% t(tuk.mat)

head(fit)

colMeans(fit)

comp <- tidyMCMC(as.mcmc(fit), conf.int=TRUE,conf.method='HPDinterval')

(MontiporaButterfly <- ggplot(comp, aes(y=estimate, x=term)) +

geom_pointrange(aes(ymin=conf.low, ymax=conf.high)) +

geom_hline(yintercept=0, linetype='dashed') +

#scale_x_continuous(breaks = c(0.5, 1, 1.5))

coord_flip() +

theme_classic()+

ggtitle(''))

#Specific questions:

newdata = with(coral, expand.grid(Year = levels(Year)))

Xmat = model.matrix(~Year, newdata)

coefs = as.data.frame(coral.stanM) %>% dplyr:::select(starts_with('(Intercept)'), starts_with('Year')) %>% as.matrix()

fit = binomial()$linkinv(coefs %*% t(Xmat))

newdata = newdata %>% cbind(tidyMCMC(fit, conf.int = T, conf.method = 'HPDinterval'))

head(newdata)

colnames(fit)

Diff.abs <- fit[, 3] - fit[,1]

sum(Diff.abs>0)/length(Diff.abs)

tidyMCMC(as.mcmc(Diff.abs), conf.int = T, conf.method = 'HPDinterval')

Diff.perc <- (fit[,3]-fit[,1])/fit[,1]

tidyMCMC(as.mcmc(Diff.perc), conf.int = T, conf.method = 'HPDinterval')

colMeans(fit)

##################

sp <- 'Poritidae'

coral <- df %>%

filter(Category == sp) %>%

#unite(SiTr, c(Site, Transect), sep = '-') %>%

group_by(Year, Site, Transect, Category) %>%

summarise(Cover=sum(LIT)) %>%

ungroup() %>%

mutate(Year = as.factor(Year), Transect = as.factor(Transect))

coral

levels(coral$Year)

ggplot(coral, aes(Year, Cover/20, fill = Site)) + geom_boxplot()

coral <- coral %>%

group_by(Year, Site) %>%

mutate(dropit = SE(Cover) == 0) %>%

filter(dropit != 'TRUE') %>%

droplevels()

table(coral$Cover ==0)

levels(coral$Year)

coral.stanP = stan_glmer(cbind(Cover, 2000-Cover)~Year +(1|Site),

data=coral,

family='binomial',

prior = normal(1, 10),

prior_intercept = normal(0,10),

chains = 3,

iter = 3000, thin=4, warmup=1000)

stan_trace(coral.stanP)

stan_ac(coral.stanP)

posterior_vs_prior(coral.stanP, group_by_parameter = TRUE, facet_args = list(scales = "free_y"),prob = .95)

tidyMCMC(coral.stanP, conf.int = T, conf.method = 'HPDinterval')

newdata = with(coral, expand.grid(Year = levels(Year)))

Xmat = model.matrix(~Year, newdata)

coefs = as.data.frame(coral.stanP) %>% dplyr:::select(starts_with('(Intercept)'), starts_with('Year')) %>% as.matrix()

fit = binomial()$linkinv(coefs %*% t(Xmat))

newdata = newdata %>% cbind(tidyMCMC(fit, conf.int = T, conf.method = 'HPDinterval')) %>%

mutate_if(is.numeric, funs(.*100)) %>%

mutate(Year = as.numeric(as.character(Year)))

summary(newdata)

(assign(paste0(sp, 'Plot'), ggplot(newdata, aes(y=estimate, x=Year)) +

geom_linerange(aes(ymin = conf.low, ymax = conf.high)) +

geom_point() +

theme_classic()+

scale_y_continuous('Cover (%)') +

scale_x_continuous('', breaks = c(2008, 2011, 2014, 2017)) +

ggtitle(sp)

))

#Tukey's comparisons

newdata = with(coral, expand.grid(Year = levels(Year)))

Xmat = model.matrix(~Year, newdata)

coefs = as.data.frame(coral.stanP) %>% dplyr:::select(starts_with('(Intercept)'), starts_with('Year')) %>% as.matrix()

fit = binomial()$linkinv(coefs %*% t(Xmat))

tuk.mat <- contrMat(n=table(newdata$Year), type='Tukey')

fit = fit %*% t(tuk.mat)

head(fit)

colMeans(fit)

comp <- tidyMCMC(as.mcmc(fit), conf.int=TRUE,conf.method='HPDinterval')

(PoritidaeButterfly <- ggplot(comp, aes(y=estimate, x=term)) +

geom_pointrange(aes(ymin=conf.low, ymax=conf.high)) +

geom_hline(yintercept=0, linetype='dashed') +

#scale_x_continuous(breaks = c(0.5, 1, 1.5))

coord_flip() +

theme_classic()+

ggtitle(''))

#Specific questions:

newdata = with(coral, expand.grid(Year = levels(Year)))

Xmat = model.matrix(~Year, newdata)

coefs = as.data.frame(coral.stanP) %>% dplyr:::select(starts_with('(Intercept)'), starts_with('Year')) %>% as.matrix()

fit = binomial()$linkinv(coefs %*% t(Xmat))

newdata = newdata %>% cbind(tidyMCMC(fit, conf.int = T, conf.method = 'HPDinterval'))

head(newdata)

colnames(fit)

Diff.abs <- fit[, 3] - fit[,1]

sum(Diff.abs>0)/length(Diff.abs)

tidyMCMC(as.mcmc(Diff.abs), conf.int = T, conf.method = 'HPDinterval')

Diff.perc <- (fit[,3]-fit[,1])/fit[,1]

tidyMCMC(as.mcmc(Diff.perc), conf.int = T, conf.method = 'HPDinterval')

colMeans(fit)

#################

sp <- 'Faviidae'

coral <- df %>%

filter(Category == sp) %>%

#unite(SiTr, c(Site, Transect), sep = '-') %>%

group_by(Year, Site, Transect, Category) %>%

summarise(Cover=sum(LIT)) %>%

ungroup() %>%

mutate(Year = as.factor(Year), Transect = as.factor(Transect))

coral

levels(coral$Year)

ggplot(coral, aes(Year, Cover/20, fill = Site)) + geom_boxplot()

coral <- coral %>%

group_by(Year, Site) %>%

mutate(dropit = SE(Cover) == 0) %>%

filter(dropit != 'TRUE') %>%

droplevels()

table(coral$Cover ==0)

levels(coral$Year)

coral.stanF = stan_glmer(cbind(Cover, 2000-Cover)~Year +(1|Site),

data=coral,

family='binomial',

prior = normal(1, 10),

prior_intercept = normal(0,10),

chains = 3,

iter = 3000, thin=4, warmup=1000)

stan_trace(coral.stanF)

stan_ac(coral.stanF)

posterior_vs_prior(coral.stanF, group_by_parameter = TRUE, facet_args = list(scales = "free_y"),prob = .95)

tidyMCMC(coral.stanF, conf.int = T, conf.method = 'HPDinterval')

newdata = with(coral, expand.grid(Year = levels(Year)))

Xmat = model.matrix(~Year, newdata)

coefs = as.data.frame(coral.stanF) %>% dplyr:::select(starts_with('(Intercept)'), starts_with('Year')) %>% as.matrix()

fit = binomial()$linkinv(coefs %*% t(Xmat))

newdata = newdata %>% cbind(tidyMCMC(fit, conf.int = T, conf.method = 'HPDinterval')) %>%

mutate_if(is.numeric, funs(.*100)) %>%

mutate(Year = as.numeric(as.character(Year)))

summary(newdata)

(assign(paste0(sp, 'Plot'), ggplot(newdata, aes(y=estimate, x=Year)) +

geom_linerange(aes(ymin = conf.low, ymax = conf.high)) +

geom_point() +

theme_classic()+

scale_y_continuous('Cover (%)') +

scale_x_continuous('', breaks = c(2008, 2011, 2014, 2017)) +

ggtitle(sp)

))

#Tukey's comparisons

newdata = with(coral, expand.grid(Year = levels(Year)))

Xmat = model.matrix(~Year, newdata)

coefs = as.data.frame(coral.stanF) %>% dplyr:::select(starts_with('(Intercept)'), starts_with('Year')) %>% as.matrix()

fit = binomial()$linkinv(coefs %*% t(Xmat))

tuk.mat <- contrMat(n=table(newdata$Year), type='Tukey')

fit = fit %*% t(tuk.mat)

head(fit)

colMeans(fit)

comp <- tidyMCMC(as.mcmc(fit), conf.int=TRUE,conf.method='HPDinterval')

(FaviidaeButterfly <- ggplot(comp, aes(y=estimate, x=term)) +

geom_pointrange(aes(ymin=conf.low, ymax=conf.high)) +

geom_hline(yintercept=0, linetype='dashed') +

#scale_x_continuous(breaks = c(0.5, 1, 1.5))

coord_flip() +

theme_classic()+

ggtitle(''))

#Specific questions:

newdata = with(coral, expand.grid(Year = levels(Year)))

Xmat = model.matrix(~Year, newdata)

coefs = as.data.frame(coral.stanF) %>% dplyr:::select(starts_with('(Intercept)'), starts_with('Year')) %>% as.matrix()

fit = binomial()$linkinv(coefs %*% t(Xmat))

newdata = newdata %>% cbind(tidyMCMC(fit, conf.int = T, conf.method = 'HPDinterval'))

head(newdata)

colnames(fit)

Diff.abs <- fit[, 3] - fit[,1]

sum(Diff.abs>0)/length(Diff.abs)

tidyMCMC(as.mcmc(Diff.abs), conf.int = T, conf.method = 'HPDinterval')

Diff.perc <- (fit[,3]-fit[,1])/fit[,1]

tidyMCMC(as.mcmc(Diff.perc), conf.int = T, conf.method = 'HPDinterval')

colMeans(fit)

#################

sp <- 'Other HC'

coral <- df %>%

filter(Category == sp) %>%

#unite(SiTr, c(Site, Transect), sep = '-') %>%

group_by(Year, Site, Transect, Category) %>%

summarise(Cover=sum(LIT)) %>%

ungroup() %>%

mutate(Year = as.factor(Year), Transect = as.factor(Transect))

coral

levels(coral$Year)

ggplot(coral, aes(Year, Cover/20, fill = Site)) + geom_boxplot()

coral <- coral %>%

group_by(Year, Site) %>%

mutate(dropit = SE(Cover) == 0) %>%

filter(dropit != 'TRUE') %>%

droplevels()

table(coral$Cover ==0)

levels(coral$Year)

coral.stanO = stan_glmer(cbind(Cover, 2000-Cover)~Year +(1|Site),

data=coral,

family='binomial',

prior = normal(1, 10),

prior_intercept = normal(0,10),

chains = 3,

iter = 3000, thin=4, warmup=1000)

stan_trace(coral.stanO)

stan_ac(coral.stanO)

posterior_vs_prior(coral.stanO, group_by_parameter = TRUE, facet_args = list(scales = "free_y"),prob = .95)

tidyMCMC(coral.stanO, conf.int = T, conf.method = 'HPDinterval')

newdata = with(coral, expand.grid(Year = levels(Year)))

Xmat = model.matrix(~Year, newdata)

coefs = as.data.frame(coral.stanO) %>% dplyr:::select(starts_with('(Intercept)'), starts_with('Year')) %>% as.matrix()

fit = binomial()$linkinv(coefs %*% t(Xmat))

newdata = newdata %>% cbind(tidyMCMC(fit, conf.int = T, conf.method = 'HPDinterval')) %>%

mutate_if(is.numeric, funs(.*100)) %>%

mutate(Year = as.numeric(as.character(Year)))

summary(newdata)

(OtherHCPlot <- ggplot(newdata, aes(y=estimate, x=Year)) +

geom_linerange(aes(ymin = conf.low, ymax = conf.high)) +

geom_point() +

theme_classic()+

scale_y_continuous('Cover (%)') +

scale_x_continuous('', breaks = c(2008, 2011, 2014, 2017)) +

ggtitle(sp))

#Tukey's comparisons

newdata = with(coral, expand.grid(Year = levels(Year)))

Xmat = model.matrix(~Year, newdata)

coefs = as.data.frame(coral.stanO) %>% dplyr:::select(starts_with('(Intercept)'), starts_with('Year')) %>% as.matrix()

fit = binomial()$linkinv(coefs %*% t(Xmat))

tuk.mat <- contrMat(n=table(newdata$Year), type='Tukey')

fit = fit %*% t(tuk.mat)

head(fit)

colMeans(fit)

comp <- tidyMCMC(as.mcmc(fit), conf.int=TRUE,conf.method='HPDinterval')

(OtherHCButterfly <- ggplot(comp, aes(y=estimate, x=term)) +

geom_pointrange(aes(ymin=conf.low, ymax=conf.high)) +

geom_hline(yintercept=0, linetype='dashed') +

#scale_y_continuous( breaks=c(-0.005, 0, 0.005)) +

coord_flip() +

theme_classic()+

ggtitle(''))

#Specific questions:

newdata = with(coral, expand.grid(Year = levels(Year)))

Xmat = model.matrix(~Year, newdata)

coefs = as.data.frame(coral.stanO) %>% dplyr:::select(starts_with('(Intercept)'), starts_with('Year')) %>% as.matrix()

fit = binomial()$linkinv(coefs %*% t(Xmat))

newdata = newdata %>% cbind(tidyMCMC(fit, conf.int = T, conf.method = 'HPDinterval'))

head(newdata)

colnames(fit)

Diff.abs <- fit[, 3] - fit[,1]

sum(Diff.abs>0)/length(Diff.abs)

tidyMCMC(as.mcmc(Diff.abs), conf.int = T, conf.method = 'HPDinterval')

Diff.perc <- (fit[,3]-fit[,1])/fit[,1]

tidyMCMC(as.mcmc(Diff.perc), conf.int = T, conf.method = 'HPDinterval')

colMeans(fit)

################

modelled <- grid.arrange(AcroporaPlot, AcroporaButterfly, MontiporaPlot, MontiporaButterfly, FaviidaePlot, FaviidaeButterfly, PoritidaePlot, PoritidaeButterfly, OtherHCPlot, OtherHCButterfly, nrow = 5)

#ggsave('modelled.pdf', modelled, height = 12, width = 6, unit = 'in')

#ggsave('modelled.png', modelled, height = 12, width = 6, unit = 'in')

################

#overall coral cover

levels(df$Category)

coral <- df %>%

filter(!Category %in% c('Non-HC', 'Soft corals')) %>%

#unite(SiTr, c(Site, Transect), sep = '-') %>%

group_by(Year, Site, Transect) %>%

summarise(Cover=sum(LIT)) %>%

ungroup() %>%

mutate(Year = as.factor(Year), Transect = as.factor(Transect))

coral

levels(coral$Year)

ggplot(coral, aes(Year, Cover/20, fill = Site)) + geom_boxplot()

coral <- coral %>%

group_by(Year, Site) %>%

mutate(dropit = SE(Cover) == 0) %>%

filter(dropit != 'TRUE') %>%

droplevels()

table(coral$Cover ==0)

levels(coral$Year)

#leave out no-variance timepoints, as stats cannot be calculated on no vairance. use % instead of proportions, cause bayesian bionmial won't run on non-integers. Keep sites as random effects, otherwise its pseudo-repliaction (transets dont represent teh reef, they represent the sites, and the sites represent the reef).

#bayesian:

coral.stanT = stan_glmer(cbind(Cover, 2000-Cover)~Year +(1|Site),

data=coral,

family='binomial',

prior = normal(1, 10),

prior_intercept = normal(0,10),

chains = 3,

iter = 3000, thin=4, warmup=1000)

stan_trace(coral.stanT)

stan_ac(coral.stanT)

posterior_vs_prior(coral.stanT, group_by_parameter = TRUE, facet_args = list(scales = "free_y"),prob = .95)

d <- tidyMCMC(coral.stanT, conf.int = T, conf.method = 'HPDinterval')

newdata = with(coral, expand.grid(Year = levels(Year)))

Xmat = model.matrix(~Year, newdata)

coefs = as.data.frame(coral.stanT) %>% dplyr:::select(starts_with('(Intercept)'), starts_with('Year')) %>% as.matrix()

fit = binomial()$linkinv(coefs %*% t(Xmat))

newdata = newdata %>% cbind(tidyMCMC(fit, conf.int = T, conf.method = 'HPDinterval')) %>%

mutate_if(is.numeric, funs(.*100)) %>%

mutate(Year = as.numeric(as.character(Year)))

summary(newdata)

ggplot(newdata, aes(y=estimate, x=Year)) +

geom_linerange(aes(ymin = conf.low, ymax = conf.high)) +

geom_point() +

theme_classic()+

scale_y_continuous('Cover (%)') +

scale_x_continuous('', breaks = c(2008, 2011, 2014, 2017))

head(fit)

sum(fit[, 1]-(fit[,4])<0)/nrow(fit)

colMeans(fit)

#Tukey's comparisons

library(multcomp)

newdata = with(coral, expand.grid(Year = levels(Year)))

Xmat = model.matrix(~Year, newdata)

coefs = as.data.frame(coral.stanT) %>% dplyr:::select(starts_with('(Intercept)'), starts_with('Year')) %>% as.matrix()

fit = binomial()$linkinv(coefs %*% t(Xmat))

tuk.mat <- contrMat(n=table(newdata$Year), type='Tukey')

fit = fit %*% t(tuk.mat)

head(fit)

colMeans(fit)

comp <- tidyMCMC(as.mcmc(fit), conf.int=TRUE,conf.method='HPDinterval')

ggplot(comp, aes(y=estimate, x=term)) +

geom_pointrange(aes(ymin=conf.low, ymax=conf.high)) +

geom_hline(yintercept=0, linetype='dashed') +

#scale_x_continuous(breaks = c(0.5, 1, 1.5))

coord_flip() +

theme_classic()

sum(fit[,3]>0)/nrow(fit)

########################

########################

# Quadrat data

df <- read.csv('quadrats.csv', strip.white=T)

#quick descriptive stats

qds <- df %>% mutate(mD = (D1+D2)/2) %>%

filter(mD >=1) %>%

group_by(Year) %>%

summarise(count = n())

newdata <- df %>%

filter(Category %in% c('Acropora', 'Montipora', 'Poritidae', 'Pocilloporidae', 'Faviidae')) %>%

mutate(mD = (D1+D2)/2,

bin = cut(mD, breaks = c(0, 1, 2, 3, 4, 5, 6, 7, 8, 9, 10, 15, 20, 25, 30, 40, 50, 999 ))) %>%

group_by(Year, Quadrat, Category, bin) %>%

summarise(Coral.Density=n()/mean(Qarea)) %>%

spread(bin, Coral.Density) %>%

gather(bin, Coral.Density, 4:19) %>%

mutate(Coral.Density = ifelse(is.na(Coral.Density), 0, Coral.Density)) %>%

group_by(Year, Category, bin) %>%

summarise(Mean.Dens=mean(Coral.Density), SE.Dens=SE(Coral.Density)) %>%

mutate(bin = as.factor(gsub(',', '', str_sub(bin,2,3))),

lower = Mean.Dens-SE.Dens, upper = Mean.Dens+SE.Dens)

levels(newdata$bin)

newdata$bin <- factor(newdata$bin, levels = c("0", "1", "2", "3", "4", "5", "6", "7", "8", "9", "10", "15", "20", "25", "30", "40"), labels = c("<1", "1-2", "2-3", "3-4", "4-5", "5-6", "6-7", "7-8", "8-9", "9-10", "10-15", "15-20", "20-25", "25-30", "30-40", "40+"))

levels(newdata$Category)

newdata$Category <- factor(newdata$Category, levels = c("Acropora", "Montipora", "Pocilloporidae", "Faviidae", "Poritidae"))

newdata$Year <-as.factor(newdata$Year)

summary(newdata)

head(newdata)

newdata <- newdata %>% filter(bin != "<1")

#let's plot it

ggplot(newdata, aes(bin, Mean.Dens)) +

geom_col(fill = 'black') +

geom_errorbar(aes(ymin = lower, ymax = upper), width = 0, col = 'gray50') +

#geom_pointrange(aes(ymin = (Mean.Dens - SE.Dens), ymax = (Mean.Dens + SE.Dens))) +

facet_grid(Year~Category) +

theme_bw() +

theme(panel.grid.major = element_blank(), panel.grid.minor = element_blank()) +

theme(axis.text.x = element_text(angle = 90, hjust=1,vjust=0.5)) +

scale_x_discrete(expression(Colony~diameter~(cm))) +

scale_y_continuous(expression(Mean~colony~density~(m^-2)), expand = c(0,0))

#ggsave('recruitment3.pdf', width = 30, height = 15, units = 'cm')

####################

#Figs of density, cover and diversity - raw data

newdata2 <- df %>%

group_by(Year, Depth, Quadrat) %>%

summarise(percent.cover=sum(Area/100)/mean(Qarea)) %>%

group_by(Year, Depth) %>%

summarise(Cov.Mean=mean(percent.cover), Cov.SE=SE(percent.cover))

newdata2$Year <- as.factor(newdata2$Year)

head(newdata2)

(p1 <- ggplot(newdata2, aes(y=Cov.Mean, x=Year, group = Depth, fill = Depth)) +

geom_linerange(aes(ymax=Cov.Mean+Cov.SE, ymin=Cov.Mean-Cov.SE, color = Depth), position = position_dodge(0.2), size = 0.3, alpha = 0.5) +

geom_line(position = position_dodge(0.2), aes(linetype = Depth)) +

geom_point(position = position_dodge(0.2), shape = 21, size = 4) +

theme_classic(12) +

scale_y_continuous(expression(Mean~('+-'~SE)~'%'~coral~cover)) +

scale_x_discrete('') +

theme(legend.position = 'NULL'))

newdata3 <- df %>%

group_by(Year, Depth, Quadrat) %>%

summarise(Coral.Density=n()/mean(Qarea)) %>%

group_by(Year, Depth) %>%

summarise(Mean.Dens=mean(Coral.Density), SE.Dens=SE(Coral.Density))

newdata3$Year <- as.factor(newdata3$Year)

(p2 <- ggplot(newdata3, aes(y=Mean.Dens, x=Year, group = Depth, fill = Depth)) +

geom_linerange(aes(ymax=Mean.Dens+SE.Dens, ymin=Mean.Dens-SE.Dens, color = Depth), position = position_dodge(0.2), size = 0.3, alpha = 0.5) +

geom_line(position = position_dodge(0.2), aes(linetype = Depth)) +

geom_point(position = position_dodge(0.2), shape = 21, size = 4) +

theme_classic(12) +

scale_y_continuous(expression(Mean~('+-'~SE)~colony~density~'/'~m^2)) +

scale_x_discrete('') +

theme(legend.position = c(0.35,0.8), legend.key.width = unit(2, 'lines')))

newdata4 <- df %>%

group_by(Year, Depth, Quadrat) %>%

summarise(Coral.Diversity=n_distinct(Genus)*25/mean(Qarea)) %>%

group_by(Year, Depth) %>%

summarise(Mean.Div=mean(Coral.Diversity), SE.Div=SE(Coral.Diversity))

newdata4$Year <- as.factor(newdata4$Year)

(p3 <- ggplot(newdata4, aes(y=Mean.Div, x=Year, group = Depth, fill = Depth)) +

geom_linerange(aes(ymax=Mean.Div+SE.Div, ymin=Mean.Div-SE.Div, color = Depth), position = position_dodge(0.2), size = 0.3, alpha = 0.5) +

geom_line(position = position_dodge(0.2), aes(linetype = Depth)) +

geom_point(position = position_dodge(0.2), shape = 21, size = 4) +

theme_classic(12) +

scale_y_continuous(expression(paste('Mean (+- SE) number \n of coral genera / 25 m'^2))) +

scale_x_discrete('') +

theme(legend.position = 'NULL'))

library(gridExtra)

pG <- grid.arrange(p2, p1, p3, nrow = 1)

#ggsave(plot = pG, 'crest vs slope.pdf', width = 7.2, height = 3.1, units = 'in')

####################

####################

#stats

#DENSITY is counts, so poisson/NB

dens <- df %>%

group_by(Year, Depth, Quadrat) %>%

summarise(CC=n()/mean(Qarea)) %>%

ungroup() %>%

mutate(Year = as.factor(Year), Quadrat = as.factor(Quadrat), CC = round(round(CC, 1)*25,0)) %>%

droplevels()

summary(dens)

ggplot(dens, aes(Year, CC, color = Depth)) + geom_boxplot()

#increasing variance with mean. Poisson overinflated, so NB

library(tidyverse)

library(rstanarm)

library(broom)

library(rstan)

library(coda)

library(bayesplot)

dens.stan1 <- stan_glmer(CC~Year+Depth + (1|Quadrat), data=dens,

family='neg_binomial_2',

prior=normal(0,1),

prior_intercept=normal(0,10),

prior_aux=cauchy(0,5),

chains=3, iter=5000, warmup=2000, thin=4)

dens.stan2 <- stan_glmer(CC~Year*Depth + (1|Quadrat), data=dens,

family='neg_binomial_2',

prior=normal(0,10),

prior_intercept=normal(0,10),

prior_aux=cauchy(0,5),

chains=3, iter=5000, warmup=2000, thin=4)

#dens.stan3 <- stan_glmer(CC~Year+Depth + (Depth|Quadrat), data=dens,

# family='neg_binomial_2',

# prior=normal(0,1),

# prior_intercept=normal(0,10),

# prior_aux=cauchy(0,5),

# chains=3, iter=5000, warmup=2000, thin=4)

#dens.stan4 <- stan_glmer(CC~Year*Depth + (Depth|Quadrat), data=dens,

# family='neg_binomial_2',

# prior=normal(0,1),

# prior_intercept=normal(0,10),

# prior_aux=cauchy(0,5),

# chains=3, iter=5000, warmup=2000, thin=4)

l1=loo(dens.stan1, k_threshold = 0.7)

l2=loo(dens.stan2, k_threshold = 0.7)

#l3=loo(dens.stan3, k_threshold = 0.7)

#l4=loo(dens.stan4, k_threshold = 0.7)

compare_models(l1,l2)

#Overdispersion (for Poisson)

#y <- dens.stan2$y #extract raw fitted values

#mu <- fitted(dens.stan2)

#r <- poisson()$dev.resids(y, mu, wt = 1)

#res <- sqrt(pmax(r, 0))

#resid <- ifelse(y > mu, res, -res)

#RSS <- sum(resid^2)

#RSS/(nrow(dens) - length(coef(dens.stan2)) - 1)

stan_ac(dens.stan2)

stan_trace(dens.stan2)

posterior_vs_prior(dens.stan2, color_by='vs', group_by=TRUE, facet_args=list(scales='free_y'))

pp_check(dens.stan1, group = interaction(dens$Year, dens$Depth), 'stat_grouped')

#everything looks good, so let's summarize

a<-tidyMCMC(dens.stan2$stanfit,conf.int=TRUE, conf.level=0.95, conf.method = 'HPDinterval', rhat = T)

plot(dens.stan1)

plot(dens.stan2)

#Lets do our summary plot, and then we will work out what posthoc tests make sense.

newdata = with(dens, expand.grid(Year = levels(Year), Depth = levels(Depth)))

Xmat = model.matrix(~Year*Depth, newdata)

colnames(as.data.frame(dens.stan2))

coefs = as.matrix(dens.stan2)[,1:6]

head(coefs)

fit = exp(coefs %*% t(Xmat))

newdata = cbind(newdata, data.frame(Mean=apply(fit, 2, mean),

lower=apply(fit,2,quantile,p=0.025),

upper=apply(fit,2,quantile,p=0.975)))

p1 <- ggplot(newdata, aes(as.numeric(as.character(Year)), Mean)) +

geom_line(aes(linetype = Depth), position = position_dodge(0.2)) +

geom_linerange(aes(ymin=lower, ymax=upper, color = Depth), position = position_dodge(0.2)) +

geom_point(aes(fill = Depth), position = position_dodge(0.2), shape = 21, size = 5) +

theme_classic() +

scale_y_continuous(expression(Number~of~colonies ~'/'~25~m^2)) +

scale_x_continuous('', breaks =c(2011, 2013, 2015)) +

theme(legend.position = c(0.2,0.9), legend.title = element_blank(), legend.key.width = unit(2, 'lines'))

p1

dens.glmmPQL <- glmmPQL(CC ~ Year*Depth, random = ~1|Quadrat, data = dens, family = 'poisson')

summary(dens.glmmPQL)

library(effects)

plot(allEffects(dens.glmmPQL, transformation = list(link = log, inverse = exp)), multiline = T, ci.style = 'bars')

newdata = with(dens, expand.grid(Year = levels(Year), Depth = levels(Depth)))

Xmat = model.matrix(~Year*Depth, newdata)

Xmat.crest <- Xmat[newdata$Depth=="Crest",]

Xmat.slope <- Xmat[newdata$Depth=="Slope",]

Xmat.dif <- Xmat.crest-Xmat.slope

#rownames(Xmat.dif) <- levels(copper$)

fit.c = coefs %*% t(Xmat.crest)

fit.s = coefs %*% t(Xmat.slope)

fit.a = exp(fit.c) - exp(fit.s) #absolute

comp.a <- tidyMCMC(fit.a, conf.int=TRUE, conf.method='HPDinterval') %>%

mutate(term = levels(newdata$Year))

p2 <- ggplot(comp.a, aes(y=estimate, x=term)) +

geom_pointrange(aes(ymin=conf.low, ymax=conf.high)) +

geom_hline(yintercept=0, linetype='dashed') +

#scale_x_continuous(breaks = c(0.5, 1, 1.5))

coord_flip() +

theme_classic()

#What is the probability of crest or slope being higher in different years?

dens2011 <- sum(fit.a[, 1]>0)/nrow(fit)

dens2013 <- sum(fit.a[, 2]>0)/nrow(fit)

dens2015 <- sum(fit.a[, 3]>0)/nrow(fit)

#how much faster is the net recruitment on the crest vs the slope between 2011 and 2015

dif.c <- fit.c[,3]-fit.c[,1]

dif.s <- fit.s[,3]-fit.s[,1]

dif.c.s <- as.data.frame(dif.c/dif.s)

tidyMCMC(dif.c.s,conf.int=TRUE, conf.level=0.95, conf.method = 'HPDinterval')

#####################################

#now lets do COVER.

cover <- df %>%

group_by(Year, Depth, Quadrat) %>%

summarise(CC=sum(Area/100)/mean(Qarea)) %>%

ungroup %>%

mutate(Year = as.factor(Year), Quadrat = as.factor(Quadrat), CC = round(CC *10)) %>%

droplevels()

head(cover)

ggplot(cover, aes(Year, CC, color = Depth)) + geom_boxplot()

cover.stan = stan_glmer(cbind(CC, 1000-CC)~Year*Depth +(1|Quadrat),

data=cover,

family='binomial',

prior = normal(0, 10),

prior_intercept = normal(0,10),

chains = 3,

iter = 3000, thin=4, warmup=1000)

stan_trace(cover.stan)

stan_ac(cover.stan)

posterior_vs_prior(cover.stan, group_by_parameter = TRUE, facet_args = list(scales = "free_y"),prob = .95)

b<-tidyMCMC(cover.stan$stanfit, conf.int = T, conf.method = 'HPDinterval', rhat = T)

plot(cover.stan)

#all looks good! FINALLY!

newdata = with(cover, expand.grid(Year = levels(Year), Depth = levels(Depth)))

Xmat = model.matrix(~Year*Depth, newdata)

coefs = as.matrix(cover.stan)[,1:6]

fit = binomial()$linkinv(coefs %*% t(Xmat))

newdata = newdata %>% cbind(tidyMCMC(fit, conf.int = T, conf.method = 'HPDinterval')) %>%

mutate_if(is.numeric, funs(.*100)) %>%

mutate(Year = as.numeric(as.character(Year)))

summary(newdata)

head(newdata)

p3 <- ggplot(newdata, aes(as.numeric(as.character(Year)), estimate)) +

geom_line(aes(linetype = Depth), position = position_dodge(0.2)) +

geom_linerange(aes(ymin=conf.low, ymax=conf.high, color = Depth), position = position_dodge(0.2)) +

geom_point(aes(fill = Depth), position = position_dodge(0.2), shape = 21, size = 5) +

theme_classic() +

scale_y_continuous(expression(Coral~cover~('%')), breaks = c(2, 4, 6, 8)) +

scale_x_continuous('', breaks =c(2011, 2013, 2015)) +

theme(legend.position = 'NULL')

newdata = with(cover, expand.grid(Year = levels(Year), Depth = levels(Depth)))

Xmat = model.matrix(~Year*Depth, newdata)

Xmat.crest <- Xmat[newdata$Depth=="Crest",]

Xmat.slope <- Xmat[newdata$Depth=="Slope",]

Xmat.dif <- Xmat.crest-Xmat.slope

#rownames(Xmat.dif) <- levels(copper$)

fit.c = coefs %*% t(Xmat.crest)

fit.s = coefs %*% t(Xmat.slope)

fit.a = binomial()$linkinv(fit.c) - binomial()$linkinv(fit.s) #absolute

fit.r = binomial()$linkinv(fit.c) / binomial()$linkinv(fit.s) # relative

comp.a <- tidyMCMC(fit.a, conf.int=TRUE, conf.method='HPDinterval') %>%

mutate(term = levels(newdata$Year))

comp.r <- tidyMCMC(fit.r, conf.int=TRUE, conf.method='HPDinterval') %>%

mutate(term = levels(newdata$Year))

#how much faster is the net growth on the crest vs the slope between 2011 and 2015

dif.c <- binomial()$linkinv(fit.c[,3])-binomial()$linkinv(fit.c[,1])

dif.s <- binomial()$linkinv(fit.s[,3])-binomial()$linkinv(fit.s[,1])

dif.c.s <- as.data.frame(dif.c/dif.s)

tidyMCMC(dif.c.s,conf.int=TRUE, conf.level=0.95, conf.method = 'HPDinterval')

p4 <- ggplot(comp.a, aes(y=estimate, x=term)) +

geom_pointrange(aes(ymin=conf.low, ymax=conf.high)) +

geom_hline(yintercept=0, linetype='dashed') +

#scale_x_continuous(breaks = c(0.5, 1, 1.5))

coord_flip() +

theme_classic()

#so then I can ask questions about probability

#the probability that crest is lower than slope in 2011 is 96% and that its the other way around in 2014 is 71%.

cov2011 <- sum(fit.a[, 1]>0)/nrow(fit)

cov2013 <- sum(fit.a[, 2]>0)/nrow(fit)

cov2015 <- sum(fit.a[, 3]>0)/nrow(fit)

##########################

#now lets do DIVERSITY

div <- df %>%

group_by(Year, Depth, Quadrat) %>%

summarise(CD=n_distinct(Genus)*25/mean(Qarea)) %>%

ungroup() %>%

mutate(Year = as.factor(Year), Quadrat = as.factor(Quadrat), CDr = round(CD, 0)) %>%

droplevels()

head(div)

ggplot(div, aes(Year, CD, color = Depth)) + geom_boxplot()

#looks pretty normal. let's try a gaussian and then a poisson and compare

div.stan1 <- stan_glmer(CDr~Year*Depth + (1|Quadrat), data=div,

family='gaussian',

prior=normal(0,10),

prior_intercept=normal(0,10),

prior_aux=cauchy(0,5),

chains=3, iter=6000, warmup=3000, thin=4)

div.stan2 <- stan_glmer(CDr~Year*Depth + (1|Quadrat), data=div,

family='poisson',

prior=normal(0,1),

prior_intercept=normal(0,10),

#prior_aux=cauchy(0,5),

chains=3, iter=6000, warmup=3000, thin=4)

stan_trace(div.stan1)

stan_ac(div.stan1)

posterior_vs_prior(div.stan1, group_by_parameter = TRUE, facet_args = list(scales = "free_y"),prob = .95)

stan_trace(div.stan2)

stan_ac(div.stan2)

posterior_vs_prior(div.stan2, group_by_parameter = TRUE, facet_args = list(scales = "free_y"),prob = .95)

l1=loo(div.stan1, k_threshold = 0.7)

l2=loo(div.stan2, k_threshold = 0.7)

compare_models(l1,l2)

c <-tidyMCMC(div.stan2$stanfit, conf.int = T, conf.method = 'HPDinterval', rhat = T)

plot(div.stan2)

#all looks good! FINALLY!

newdata = with(div, expand.grid(Year = levels(Year), Depth = levels(Depth)))

Xmat = model.matrix(~Year*Depth, newdata)

coefs = as.matrix(div.stan2)[,1:6]

fit = exp(coefs %*% t(Xmat))

newdata = newdata %>% cbind(tidyMCMC(fit, conf.int = T, conf.method = 'HPDinterval')) %>%

mutate(Year = as.numeric(as.character(Year)))

summary(newdata)

head(newdata)

p5 <- ggplot(newdata, aes(as.numeric(as.character(Year)), estimate)) +

geom_line(aes(linetype = Depth), position = position_dodge(0.2)) +

geom_linerange(aes(ymin=conf.low, ymax=conf.high, color = Depth), position = position_dodge(0.2)) +

geom_point(aes(fill = Depth), position = position_dodge(0.2), shape = 21, size = 5) +

theme_classic() +

scale_y_continuous(expression(Number~of~cora~genera~'/'~25~m^2)) +

scale_x_continuous('', breaks =c(2011, 2013, 2015)) +

theme(legend.position = 'NULL')

newdata = with(div, expand.grid(Year = levels(Year), Depth = levels(Depth)))

Xmat = model.matrix(~Year*Depth, newdata)

Xmat.crest <- Xmat[newdata$Depth=="Crest",]

Xmat.slope <- Xmat[newdata$Depth=="Slope",]

Xmat.dif <- Xmat.crest-Xmat.slope

#rownames(Xmat.dif) <- levels(copper$)

fit <- coefs %*% t(Xmat)

fit.c = coefs %*% t(Xmat.crest)

fit.s = coefs %*% t(Xmat.slope)

fit.a = binomial()$linkinv(fit.c) - binomial()$linkinv(fit.s) #absolute

comp.yr <- cbind(newdata, tidyMCMC(fit, conf.int=TRUE, conf.method='HPDinterval'))

comp.a <- tidyMCMC(fit.a, conf.int=TRUE, conf.method='HPDinterval') %>%

mutate(term = levels(newdata$Year))

p6 <- ggplot(comp.a, aes(y=estimate, x=term)) +

geom_pointrange(aes(ymin=conf.low, ymax=conf.high)) +

geom_hline(yintercept=0, linetype='dashed') +

#scale_x_continuous(breaks = c(0.5, 1, 1.5))

coord_flip() +

theme_classic()

div2011 <- sum(fit.a[, 1]>0)/nrow(fit.a)

div2013 <- sum(fit.a[, 2]>0)/nrow(fit.a)

div2015 <- sum(fit.a[, 3]>0)/nrow(fit.a)

head(fit)

sum(((fit[, 1]+fit[, 4])/2-(fit[,2]+fit[, 5])/2)<0)/nrow(fit)

sum(((fit[, 2]+fit[, 5])/2-(fit[,3]+fit[, 6])/2)<0)/nrow(fit)

pGrid <- grid.arrange(p1, p2, p3, p4, p5, p6, nrow = 3)

ggsave(plot = pGrid, 'crest vs slope modeled.pdf', width = 6, height = 8, units = 'in')

probs <- rbind(dens2011, dens2013, dens2015, cov2011, cov2013, cov2015, div2011, div2013, div2015)

write.csv(probs, 'probs.csv', row.names = T)

########################

########################

#and now let's think about the cohort comparisons.

newdata <- df %>%

filter(Category %in% c('Acropora')) %>%

mutate(mD = (D1+D2)/2,

bin = cut(mD, breaks = c(0, 1, 2, 3, 4, 5, 6, 7, 8, 9, 10, 15, 20, 25, 30, 40, 50, 999 ))) %>%

group_by(Year, Quadrat, Category, bin) %>%

summarise(Coral.Density=n()/mean(Qarea)) %>%

spread(bin, Coral.Density) %>%

gather(bin, Coral.Density, 4:19) %>%

mutate(Coral.Density = ifelse(is.na(Coral.Density), 0, Coral.Density)) %>%

mutate(bin = as.factor(gsub(',', '', str_sub(bin,2,3)))) %>%

ungroup() %>%

mutate(CD = round(Coral.Density*1000,0), Year = as.factor(Year), Quadrat = as.factor(Quadrat)) %>%

filter(bin == "1") %>%

droplevels()

head(newdata)

summary(newdata)

levels(newdata$bin)

freq.stan <- stan_glmer(CD~Year + (1|Quadrat), data=newdata,

family='neg_binomial_2',

prior=normal(3,10),

prior_intercept=normal(5,20),

prior_aux=cauchy(0,5),

chains=3, iter=2000, warmup=1000, thin=3)

stan_ac(freq.stan)

stan_trace(freq.stan)

posterior_vs_prior(freq.stan, color_by='vs', group_by=TRUE, facet_args=list(scales='free_y'))

tidyMCMC(freq.stan$stanfit, conf.int = T, conf.method = 'HPDinterval', rhat = T)

plot(freq.stan)

#all looks good! FINALLY!

newdata = with(div, expand.grid(Year = levels(Year), Depth = levels(Depth)))

Xmat = model.matrix(~Year*Depth, newdata)

coefs = as.matrix(div.stan2)[,1:6]

fit = exp(coefs %*% t(Xmat))

newdata = newdata %>% cbind(tidyMCMC(fit, conf.int = T, conf.method = 'HPDinterval')) %>%

mutate(Year = as.numeric(as.character(Year)))

summary(newdata)

head(newdata)

p5 <- ggplot(newdata, aes(as.numeric(as.character(Year)), estimate)) +

geom_line(aes(linetype = Depth), position = position_dodge(0.2)) +

geom_linerange(aes(ymin=conf.low, ymax=conf.high, color = Depth), position = position_dodge(0.2)) +

geom_point(aes(fill = Depth), position = position_dodge(0.2), shape = 21, size = 5) +

theme_classic() +

scale_y_continuous(expression(Number~of~cora~genera~'/'~25~m^2)) +

scale_x_continuous('', breaks =c(2011, 2013, 2015)) +

theme(legend.position = 'NULL')

newdata = with(div, expand.grid(Year = levels(Year), Depth = levels(Depth)))

Xmat = model.matrix(~Year*Depth, newdata)

Xmat.crest <- Xmat[newdata$Depth=="Crest",]

Xmat.slope <- Xmat[newdata$Depth=="Slope",]

Xmat.dif <- Xmat.crest-Xmat.slope

#rownames(Xmat.dif) <- levels(copper$)

fit.c = coefs %*% t(Xmat.crest)

fit.s = coefs %*% t(Xmat.slope)

fit.a = binomial()$linkinv(fit.c) - binomial()$linkinv(fit.s) #absolute

comp.a <- tidyMCMC(fit.a, conf.int=TRUE, conf.method='HPDinterval') %>%

mutate(term = levels(newdata$Year))

p6 <- ggplot(comp.a, aes(y=estimate, x=term)) +

geom_pointrange(aes(ymin=conf.low, ymax=conf.high)) +

geom_hline(yintercept=0, linetype='dashed') +

#scale_x_continuous(breaks = c(0.5, 1, 1.5))

coord_flip() +

theme_classic()

div2011 <- sum(fit.a[, 1]>0)/nrow(fit)

div2013 <- sum(fit.a[, 2]>0)/nrow(fit)

div2015 <- sum(fit.a[, 3]>0)/nrow(fit)
